# A Comparison of Partial Information Decompositions Using Data from Real and Simulated Layer 5b Pyramidal Cells

**DOI:** 10.3390/e24081021

**Published:** 2022-07-24

**Authors:** Jim W. Kay, Jan M. Schulz, William A. Phillips

**Affiliations:** 1 School of Mathematics and Statistics, University of Glasgow, Glasgow G12 8QQ, UK; 2 Department of Biomedicine, University of Basel, 4001 Basel, Switzerland; j.schulz@unibas.ch; 3 Faculty of Natural Sciences, University of Stirling, Stirling FK9 4LA, UK; w.a.phillips@stirling.ac.uk

**Keywords:** information theory, partial information decomposition, pointwise partial information decomposition, synergy, redundancy, cooperative context-sensitivity, apical amplification, apical drive, misinformation

## Abstract

Partial information decomposition allows the joint mutual information between an output and a set of inputs to be divided into components that are synergistic or shared or unique to each input. We consider five different decompositions and compare their results using data from layer 5b pyramidal cells in two different studies. The first study was on the amplification of somatic action potential output by apical dendritic input and its regulation by dendritic inhibition. We find that two of the decompositions produce much larger estimates of synergy and shared information than the others, as well as large levels of unique misinformation. When within-neuron differences in the components are examined, the five methods produce more similar results for all but the shared information component, for which two methods produce a different statistical conclusion from the others. There are some differences in the expression of unique information asymmetry among the methods. It is significantly larger, on average, under dendritic inhibition. Three of the methods support a previous conclusion that apical amplification is reduced by dendritic inhibition. The second study used a detailed compartmental model to produce action potentials for many combinations of the numbers of basal and apical synaptic inputs. Decompositions of the entire data set produce similar differences to those in the first study. Two analyses of decompositions are conducted on subsets of the data. In the first, the decompositions reveal a bifurcation in unique information asymmetry. For three of the methods, this suggests that apical drive switches to basal drive as the strength of the basal input increases, while the other two show changing mixtures of information and misinformation. Decompositions produced using the second set of subsets show that all five decompositions provide support for properties of cooperative context-sensitivity—to varying extents.

## 1. Introduction

A breakthrough in information theory happened in 2010 when Williams and Beer [1] published a method called *partial information decomposition,* which provided a framework under which the mutual information, shared between the inputs and output of a probabilistic system, could be decomposed into components that measure different aspects of the information: the unique information that each input conveys about the output; the shared information that all inputs possess regarding the output; the information that the inputs in combination have about the output. They also defined a measure of redundancy, Imin, together with a method for obtaining a partial information decomposition (PID), which is also commonly referred to as *Imin*. Several authors criticised the definition of the redundancy component in the Imin PID [2,3,4,5], thus spawning several new methods for computing a partial information decomposition. Harder and colleagues [2] defined a new measure of redundancy based on information projections, with a PID denoted here as *Iproj*, and they introduced the distinction between source redundancy and mechanistic redundancy. Griffiths and Koch [4] developed a measure of synergy, while independently, Bertschinger and colleagues focused on defining a measure of unique information and defined an optimisation approach to estimate each of the four PID components; it turned out that both approaches resulted in the same decomposition, commonly called *Ibroja*. Taking a pointwise approach, Ince [5] considered measuring redundancy at the level of each individual realisation of the probabilistic system by considering a common change in surprisal, thus creating a measure of redundancy for the system, and PID *Iccs*. James and colleagues defined a measure of unique information by using a lattice of maximum entropy distributions based on dependency constraints, with PID, *Idep*. Finn and Lizier [6] introduced a very detailed pointwise approach, with PID, *Ipm*. Niu and Quinn [7] produced a decomposition, *Iig*, based on information geometry. Makkeh and colleagues [8,9] defined a measure of shared information and PID, *Isx*. Most recently, Kolchinsky [10] used a general approach to define a measure of redundancy based on Blackwell ordering. The corresponding PID has been named *Iprec.*

The Imin, Iproj, Ibroja and Idep PIDs are guaranteed to have nonnegative components. In [7], it is claimed that Iig is a nonnegative PID, but examples of systems producing a negative estimate of redundancy were discovered in 2020. Nevertheless, this PID has given sensible results in many systems. No guarantee of non-negativity was given for the Iprec PID, and an example of negative synergy has been found. The other three methods are defined in a pointwise manner by considering information measures at the level of individual realisations and defining partial information components at this level. Pointwise PIDs can produce negative components and they are described as providing *misinformation* in such cases [11].

An important feature of PID is that it enables the shared information and synergistic information in a system to be estimated separately. This provides an advance on earlier research in which the interaction information was used to estimate synergy [12,13], but could be negative, and the three-way mutual information [14] or coinformation [15], which also could be negative, was used as an objective function in neural networks with two distinct sets of inputs from receptive and contextual fields, respectively.

Partial information decomposition has been applied to data in neuroscience, neuroimaging, neural networks and cellular automata; see, e.g., [16,17,18,19,20,21,22,23]. A major selling point of the Isx PID is that the components are differentiable, unlike other PIDs. This makes it possible to build neural networks with a particular neural goal involving PID components as the objective function [24,25]. For an overview of PID, see [26], and for an excellent tutorial, see [27].

We will provide a systematic comparison of the different methods by applying the Ibroja, Idep, Iccs, Ipm and Isx PIDs to data recorded in two different studies. Further detail and illustration of these PID methods are provided in Appendices Appendix A, Appendix B, Appendix C, Appendix D, Appendix E, Appendix F, Appendix G and Appendix H. In Appendix I, further comparisons are provided involving the Imin, Iproj, Iig and Iprec PIDs. First, we present comparisons of detailed analyses of physiological data recorded from cortical layer 5b (L5b) pyramidal neurons in a study of GABA ${}_{B}$ receptor-mediated regulation of dendro-somatic synergy [28]. The influence of GABA ${}_{B}$ receptor-mediated inhibition of the apical dendrites evoked by local application of the GABA ${}_{B}$ receptor agonist baclofen will be studied by making within-neuron paired comparisons of PID components. Secondly, we will also shed light on unique information asymmetries as revealed by the PID analyses, as well as discussing the evidence for apical amplification in the presence and absence of GABA ${}_{B}$ receptor-mediated inhibition of apical dendrites.

**Figure 1:**
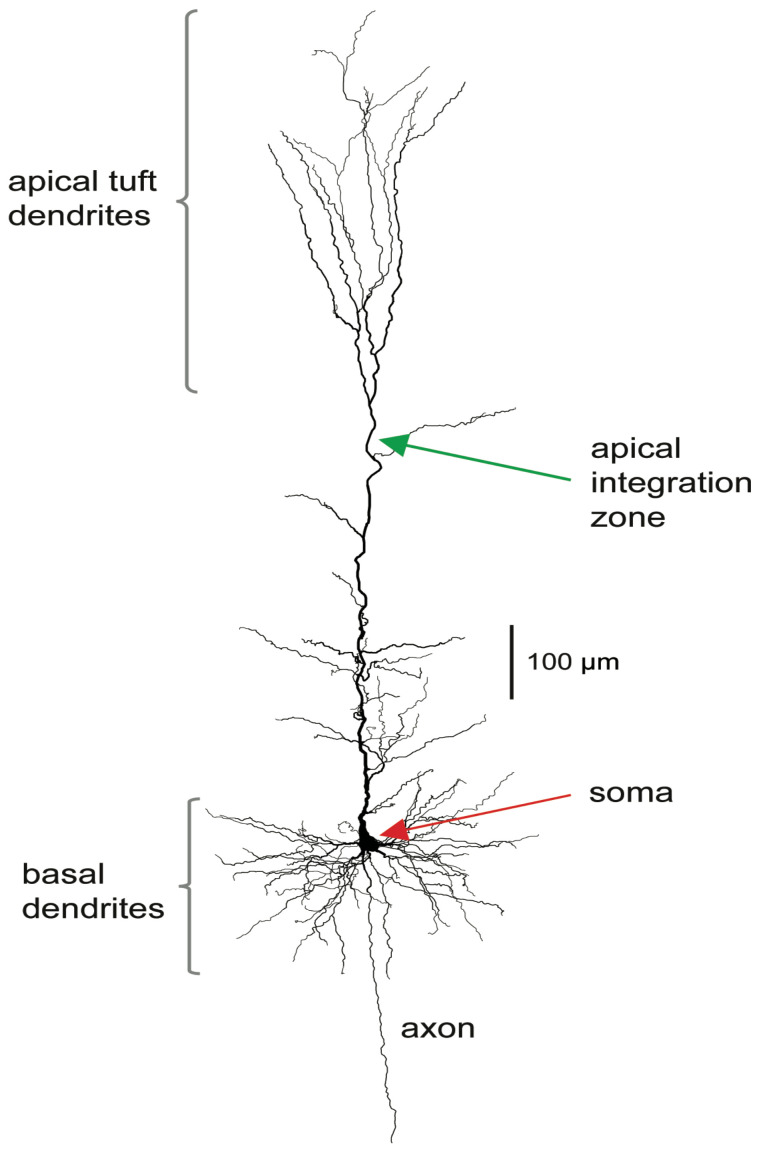
Reconstruction of a biocytin-filled L5b pyramidal neuron recorded from the rat somatosensory cortex. Basal and apical tuft dendrites are indicated. These sets of dendrites directly influence two distinct integration zones that can emit spikes: the axon initial segment close to the soma of the neuron, which initiates Na ${}^{+}$-dependent APs, and the integration zone in the apical dendrite, which initiates dendritic Ca${}^{2+}$ spikes.

The stereotypical morphology of pyramidal neurons suggests that they have at least two functionally distinct sets of fine dendrites, the basal dendrites that feed directly into the cell body, or soma, from where output action potential(AP)s are generated, and the dendrites of the apical tuft, which are far more distant from the soma and connected to it by the apical trunk. Inputs to the branches of the apical tuft arise from diverse sources that specify various aspects of the context within which the feedforward input to the basal dendrites is processed [29,30]. These apical inputs are summed at an integration zone near the top of the apical trunk (see Figure 1), which, when sufficiently activated, generates Ca${}^{2+}$-dependent regenerative potentials in the apical trunk, thus providing a cellular mechanism by which these pyramidal cells can respond more strongly to activation of their basal dendrites when that coincides with activation of their apical dendrites [31]. Though these experiments require an exceptionally high level of technical expertise, there are now many anatomical and physiological studies indicating that some classes of pyramidal cell can operate as context-sensitive two-point processors, rather than as integrate-and-fire point processors [31,32,33]. Thick-tufted L5b pyramidal cells are the class of pyramidal cell in which operational modes approximating context-sensitive two-point processing have most clearly been demonstrated, but it may apply to some other classes of pyramidal cell also, though not to all [34]. Mechanistically, these operations are supported by dendritic nonlinear integration of synaptic inputs, including dendritic Ca${}^{2+}$ spikes and the voltage-dependence of the transfer resistance from dendrite to soma [28,35]. These advances in our knowledge of the division of labor between apical and basal dendrites now give the interaction between apical and basal dendritic compartments a prominent role within the broader field of dendritic computation [36].

The second study [37] considers data on spike counts obtained using an amended version of the Hay compartmental model [38]. Spike counts are available for many different combinations of basal and apical inputs. While PIDs can be computed for the entire dataset, an interesting diversity of balance between basal drive and apical drive is revealed by applying the PID methods to subsets of the data defined by various combinations of basal and apical inputs. For one set of subsets, this reveals a bifurcation in unique information asymmetry and a difference among the methods in how this is expressed. A different analysis of subsets allows a full discussion of the extent to which evidence of cooperative context-sensitivity is revealed by the nature of the PID components.

Many empirical findings have been interpreted as indicating that cooperative context-sensitivity is common throughout perceptual and higher cognitive regions of the mammalian neocortex. For example, consider the effect of a flanking context on the ability to detect a short faint line. A surrounding context is neither necessary nor sufficient for that task, but many psychophysical and physiological studies show that context can have large effects, as reviewed, for example, by Lamme [39,40,41]. In [42,43], theoretical studies on the effects of context (then called ‘contextual modulation’) were explored. The ideal properties of cooperative context-sensitivity are described in Section 2.

The goal of this work is two-fold. First, for the datasets considered, we wish to compare the results obtained by employing the different PID methods on probability distributions defined using real and simulated data. Secondly, we intend to use the various PIDs to make inferences about the functioning of the pyramidal cells under investigation.

## 2. Methods

### 2.1. Data

Physiological data recorded from rat L5b pyramidal neurons during dual patch-clamp recordings from soma and apical dendrite before and during local application of the GABA ${}_{B}$ B receptor agonist baclofen was taken from [28]. Spike count data obtained using an amended version of the Hay compartmental model was taken from [37].

### 2.2. Notation and Definitions

We consider trivariate probabilistic systems involving three discrete random variables: an output *Y* and two inputs *B* and *A*. Hence, underlying the discrete data sets we consider a probability mass function Pr(Y=y,B=b,A=a), where y,b,a belong to the finite alphabets $A_{Y},A_{B},A_{A}$, respectively.

We now define the standard information theoretic terms that are required in this work and they are based on results in [44]. We denote by the function *H*, the usual Shannon entropy, and note that any term with zero probability makes no contribution to the sums involved. The joint mutual information that is shared by *Y* and the pair (B,A) is given by,

(1) I(Y;B,A)=H(Y)+H(B,A)−H(Y,B,A).

The information that is shared between *Y* and *B*, having taken into account the influence of *A* is:

(2) I(Y;B|A)=H(Y,A)+H(B,A)−H(A)−H(Y,B,A),

and the information that is shared between *Y* and *A* having taken into account the influence of *B* is:

(3) I(Y;A|B)=H(Y,B)+H(B,A)−H(B)−H(Y,B,A).

The information shared between *Y* and *B* is:

(4) I(Y;B)=H(Y)+H(B)−H(Y,B)

and between *Y* and *A* is:

(5) I(Y;A)=H(Y)+H(A)−H(Y,A)

The interaction information [45] is a measure of information involving all three variables, Y,B,A and is defined by:

(6) II(Y;B;A)=I(Y;B,A)−I(Y;B)−I(Y;A)

The coinformation [15] is closely linked to the interaction information, being its negative, and is given by:

(7) coi(Y;B;A)=I(Y;B)+I(Y;A)−I(Y;B,A)

### 2.3. Partial Information Decomposition

The information decomposition can be expressed as [24]:

(8) $$I\left(Y;B,A\right)=I_{unq}\left(Y;B|A\right)+I_{unq}\left(Y;A|B\right)+I_{shd}\left(Y;B,A\right)+I_{syn}\left(Y;B,A\right).$$

Adapting the notation of [24] we express our joint input mutual information in four terms as follows:
UnqB≡$I_{unq}\left(Y;B|A\right)$denotes the unique information that *B* conveys about *Y*;
UnqA≡$I_{unq}\left(Y;A|B\right)$is the unique information that *A* conveys about *Y*;
Shd≡$I_{shd}\left(Y;B,A\right)$gives the shared (or redundant) information that both *B* and *A* have about *Y*;
Syn≡$I_{syn}\left(Y;B,A\right)$is the synergy or information that the joint variable (B,A) has about *Y* that cannot be obtained by observing *B* and *A* separately.

It is possible to make deductions about a PID by using the following four equations, which give a link between the components of a PID and certain classical Shannon measures of mutual information. The following are in ([24], Equations (4) and (5)), with amended notation; see also [1].

(9) $$\begin{array}{cc} I(Y;B) & =\mathrm{Unq}B+\mathrm{Shd} \end{array}$$

(10) $$\begin{array}{cc} I(Y;A) & =\mathrm{Unq}A+\mathrm{Shd}, \end{array}$$

(11) $$\begin{array}{cc} I(Y;B|A) & =\mathrm{Unq}B+\mathrm{Syn}, \end{array}$$

(12) $$\begin{array}{cc} I(Y;A|B) & =\mathrm{Unq}A+\mathrm{Syn}. \end{array}$$

Using (8)–(10), we may deduce the following connections between classical information measures and partial information components.

(13) II(Y;B;A)=Syn−Shd

(14) I(Y;B)−I(Y;A)=UnqB−UnqA

When the partial information components are known a priori to be non-negative, we may deduce the following from (1), (9), (10). When the interaction information in (6) is positive, a lower bound on the synergy of a system is given by the interaction information [45]. When the coinformation in (7) is positive, a lower bound on the shared information of a system is given by the value of the coinformation [15]. Furthermore, the expression in (14) provides a lower bound for UnqB, when I(Y;B)>I(Y;A). Thus some deductions can be made without considering a PID. While such deductions can be useful in providing information bounds, it is only by computing a PID that the actual values of the partial information components can be obtained.

When making comparisons between different systems, it is sometimes necessary to normalise the PID components by dividing each term by their sum, the joint mutual information, I(Y;B,A). Such normalisation will be applied in the analyses considered in the sequel. This means that the sum of the PID components is equal to unity and so they are negatively correlated.

In this study, the PID component, Shd, has not been separated into sum of source, ShdS, and mechanistic, ShdM, terms [2,42,46] as:

Shd=ShdS+ShdM

because not all of the five PIDs considered include definitions regarding how to achieve this task. For probability distributions in which the inputs *B* and *A* are marginally independent, the source shared information, ShdS, should be equal to zero, and hence the shared information, Shd, is entirely mechanistic shared information—shared information due to the probabilisitic mechanism involved in the information processing.

### 2.4. Unique Information Asymmetry

We define the unique information asymmetry (UIA) to be UnqB − UnqA. From (11), (12), (14) we have that:

(15) UnqB−UnqA=I(Y;B)−I(Y;A)=I(Y;B|A)−I(Y;A|B).

The value of UIA is the same for every PID method. When UIA > 0, we say that the basal input is mainly driving, whereas when UIA < 0 it is the apical input that is mainly providing the drive. Asymmetries for which UIA > 0 and UnqA is zero or small in magnitude are of interest in relation to property CSS3 of cooperative context-sensitivity, as defined below.

### 2.5. Pointwise PID Methods

The PID methods Ibroja and Idep produce PID components that are non-negative, whereas Iccs, Ipm and Isx can produce negative values. The PIDs Iccs, Ipm and Isx are pointwise-based methods in which local information measures are employed at the level of individual realisations of the random variables. Local mutual information is explained by Lizier in [11]. If U,V are discrete random variables then the mutual information I(U,V) shared between *U* and *V* can be written as an average of the local mutual information terms i(u;v), for each individual realisation (u,v) of (U,V), as follows:

(16) $$I\left(U;V\right)=\sum_{u,v}p\left(u,v\right)\log_{2}\frac{p(u,v)}{p(u)p(v)}=\sum_{u,v}p\left(u,v\right)\log_{2}\frac{p(u|v)}{p(u)}=\sum_{u,v}p\left(u,v\right)\log_{2}i\left(u;v\right),$$

where

$$i\left(u;v\right)=\log_{2}\frac{p(u|v)}{p(u)}$$

is the local mutual information associated with the realisation (u,v) of (U,V).

The local mutual information i(u;v) is positive when p(u|v)>p(u), so that “knowing the value of *v* increased our expectation of (or positively informed us about) the value of the measurement *u*” [11]. The local mutual information i(u;v) is negative when p(u|v)<p(u), so that “knowing about the value of *v* actually changed our belief p(u) about the probability of occurrence of the outcome *u* to a smaller value p(u|v), and hence we considered it less likely that *u* would occur when knowing *v* than when not knowing *v*, in a case were *u* nevertheless occurred” [11]. Of course, the average of these local measures is the mutual information I(U;V), as in (16), but when pointwise information measures are used to construct a PID there can be negative averages. For further details of how negative values of PID components can occur, see [5,6,8,42].

In the analyses reported below, it will be found that the pointwise PIDs can give negative values for the unique information due to *A*, or for the unique information due to *B*, or both. We interpret this to mean that the unique information provided by *A*, or by *B* is, on average, less likely to result in predicting the correct value of the output *Y*. We adopt the term ‘misinformation’ from [6,8,11], and describe this as ‘unique misinformation due to *A* (or *B*)’.

### 2.6. Ideal Properties of Cooperative Context-Sensitivity

We now state key properties of cooperative context-sensitivity (which are a modified form of those specified for contextual modulation in [43]), while recognising that in any biological system these properties are likely to be observed only as an approximation to the ideal. It is assumed that the basal input is driving and the apical input provides the context. The context amplifies the transmission of information about the necessary, or driving, input when criterion CCS3 is met.

CCS1: The drive, *B*, is sufficient for the output to transmit information about the input, so context, *A*, is not necessary.
CCS2: The drive, *B*, is necessary for the output to transmit information about the input, so context, *A*, is not sufficient.
CCS3: The output transmits unique information about the drive, *B*, but little or no unique information or misinformation about the context, *A*, although synergistic or shared mechanistic components, or both, are present.
CCS4: The context strengthens the transmission of information about *B* when *B* is weak. As the strength of *B* increases, the synergy and shared mechanistic information decrease.

### 2.7. Statistics

Summary statistics are presented as the sample median and the sample quartiles. Significance testing based on within-neuron differences is conducted using a two-sided exact Wilcoxon signed rank test of equality of population medians, and the threshold for declaring statistical significance of a single test is P < 0.05. Where multiple tests are used, the individual *p*-values were corrected by using the Bonferroni method to ensure that the family-wise error rate is at most 0.05; if *m* simultaneous tests are conducted, a test with *p*-value P has a corrected value of min(mP,1).

### 2.8. Software

The Ibroja PID was estimated using compute UI [47]. The discrete information theory library dit [48] was used to estimate the Imin, Iproj, Iccs, Idep and Ipm PIDs. R [49] code was also used to estimate the Iig, Iccs and Idep PIDs. An amended version of the Iprec redundancy measure was provided by Artemy Kolchinsky [50]. Python code was called from RStudio [49] by using the reticulate package [51]. The graphics were produced by using the ggplot2 package [52] in RStudio. Statistical testing made use of the coin package [53] in RStudio.

## 3. Results

### 3.1. Real Data from Patch-Clamp Recordings in L5b Pyramidal Neurons

**Figure 2:**
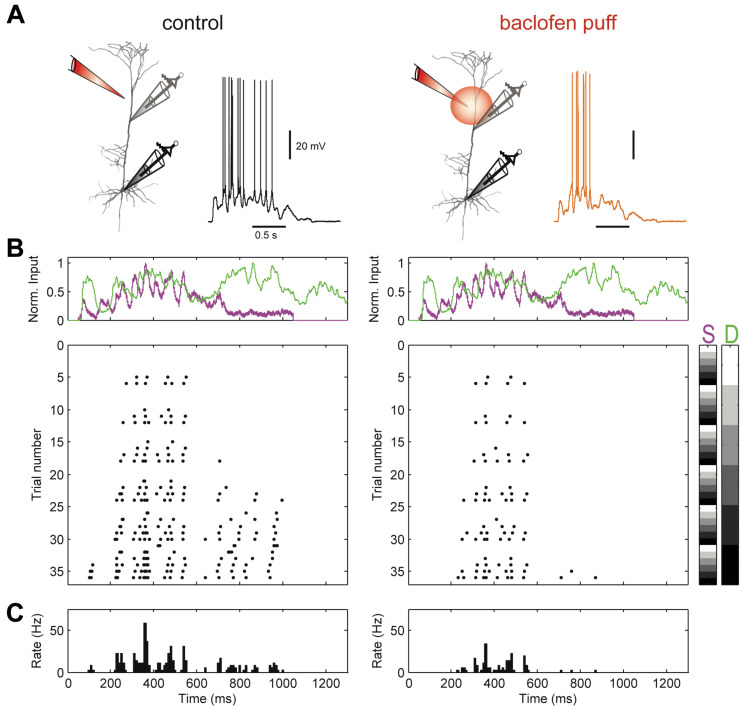
Dual dendritic and somatic patch-clamp recording from a L5b pyramidal neuron of rat somatosensory cortex enables the study of amplification of somatic AP output by apical dendritic input and its regulation by dendritic inhibition. (**A**) Locations of dual dendritic and somatic patch-clamp recordings are indicated on a biocytin-filled L5 pyramidal neuron. After recordings in the control condition, the GABA ${}_{B}$ agonist baclofen (50 μM) was puffed onto the apical dendrite at 50 to 100 μM distal to the dendritic patch pipette. Example membrane potential responses to combined current injections into soma and dendrite are shown in control condition (**left**) and during the puff of baclofen (**right**). Peak current amplitude was 1000 pA for dendritic and somatic current injections. (**B**) Top, injected current waveforms based on in vivo responses to sensory stimulation [54]. Dendritic current is shown in green, somatic in purple. Bottom, raster plot of APs emitted in individual episodes during increasing levels of dendritic and somatic stimulation strength. Control is shown on the left. A raster plot of APs emitted in the same neuron during activation of dendritic GABA ${}_{B}$ Rs by a puff of baclofen onto the apical dendrite is shown on the right. Different levels of the injected current in 36 combinations are indicated by the right colour bars (S, somatic; D, dendritic). The peak amplitude of the current waveform was increased from 0 pA (white) to 1250 (black) in soma and dendrite, respectively. Step size was 250 pA. (**C**) Peri-stimulus time histogram of APs across all current combinations for both conditions. All data were obtained from [28].

In a study of GABA ${}_{B}$ receptor-mediated regulation of dendro-somatic synergy in L5b pyramidal neurons [28], the relationship between AP output, *Y*, to input currents during combined current injections into the soma, *S* (referred to as basal input, *B*, in the sequel) and distant apical dendrite of thick-tufted L5b pyramidal neurons, *D* (referred to as apical input, *A*, in the sequel) in rat somatosensory cortex was recorded. Current waveforms injected via patch-clamp pipettes were used to mimic synaptic responses to contralateral hind limb stimulation in vivo [54]. AP trains were recorded for ≥25 (range: 25–49) combinations of different current levels (see Figure 2).

The normalised injected waveforms were scaled by separate amplification factors ranging from 0 pA up to 1500 pA, resulting in, at most, 49 trials for each neuron. Trials for which there were no APs for a treatment condition were omitted from consideration. Care was taken to ensure that the input distributions for the treatment conditions considered within a neuron contained exactly the same combinations of somatic and dendritic amplitude. This is particularly important since there is interest in comparing the PIDs obtained under different treatment conditions within each neuron. Ensuring that the input (B,A) distributions match ensures that any observed difference in a PID component within a neuron is not simply due to a difference in the input distributions. Data of time-varying input currents and resulting AP times, from the admitted trials, were binned into non-overlapping segments of 120 ms to maximise the joint mutual information (see [28], Figure S1). Within each bin, the AP number, mean somatic and mean dendritic signals were computed. The values of each of the input signals were binned into quartiles to maximise entropy [27]. The output was categorised as 0, 1 or 2+ APs. Thus, we generated a 4 by 4 by 3 probability distribution for each of the neurons considered under each of the treatment conditions.

#### 3.1.1. Classic Mutual Information Measures

**Figure 3:**
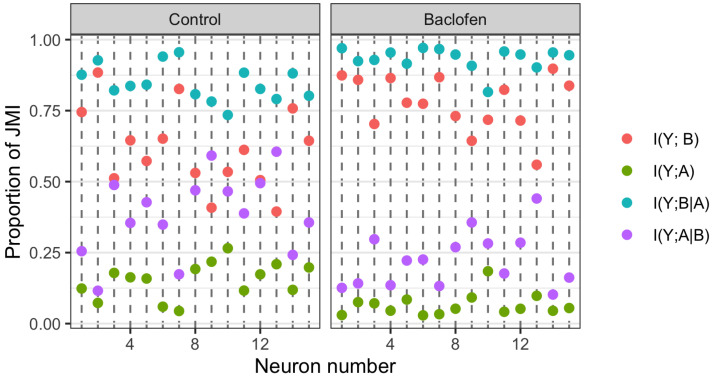
Physiological L5b neuronal recording data. For each of the 15 neurons under each of the Control and Baclofen conditions, the values of the four normalised classic mutual information measures that are involved in the definition of a PID are displayed. Their values are given as their relative contributions to the joint mutual information in each case.

The classic mutual information measures were computed for each neuron under each of the two experimental conditions. The values of the joint mutual information (JMI) between the AP count (*Y*) and the pair of basal and apical inputs (B,A) ranged from 0.49 to 0.93 bit for neurons in the Control condition and from 0.45 to 1.02 bit for neurons exposed to baclofen. Therefore, the information measures computed for each neuron under each condition were normalised by dividing by their respective JMI values. Normalised values are displayed in Figure 3. It is worth noting that, when normalised, the information measure values satisfy the equations:

(17) I(Y;B)+I(Y;A|B)=1=I(Y;A)+I(Y;B|A)

which means that I(Y;B) and I(Y;A|B) are negatively correlated, as are I(Y;A) and I(Y;B|A).

Following the vertical dashed lines in Figure 3, we notice that the mutual information between the AP count and the basal input is much larger than that between the AP count and the apical input, indicating clear unique information asymmetry. For these neurons, the AP count is more strongly related to the basal than the apical input. This is the case for both of the experimental conditions. We can, therefore, anticipate that each of the PIDs considered will also express these asymmetries between the values of their unique basal and apical PID components.

In Figure 3, for most neurons under both conditions, we see that when I(Y;B|A) is high, the corresponding I(Y;A) values are low and when the I(Y;B) values are large, the corresponding I(Y;A|B) values are small. These simply reflect the negative correlations between the respective normalised measures as a result of (17).

From Table 1, we find that I(Y;B) increases on average when baclofen is present and that the sample distribution of values has shifted upwards while having approximately the same interquartile range. It is also noticeable that I(Y;A) has decreased on average in the presence of baclofen and that the sample distribution of values has shifted downwards with an approximate 50% reduction in interquartile range. By considering (17), we find corresponding changes in the conditional mutual information I(Y;B|A) and I(Y;A|B), which indicate, on average, an increase in the conditional dependence between the AP output and the basal input along with a decrease in the conditional dependence between the AP output and the apical input. These changes, which are associated with the presence of baclofen, show that, as expected, when there is an inhibitory input to the distal apical dendrite, the AP output becomes more strongly related to the basal input and less strongly related to the apical input. It remains to be seen just how these changes are reflected in the differences between the components of the PIDs.

All thirty values of the interaction information are positive. Thus, we can deduce the presence of at least some synergy a priori for all fifteen neurons under each condition for PIDs that are guaranteed to possess nonnegative components.

#### 3.1.2. Comparison of PID Components

**Figure 4:**
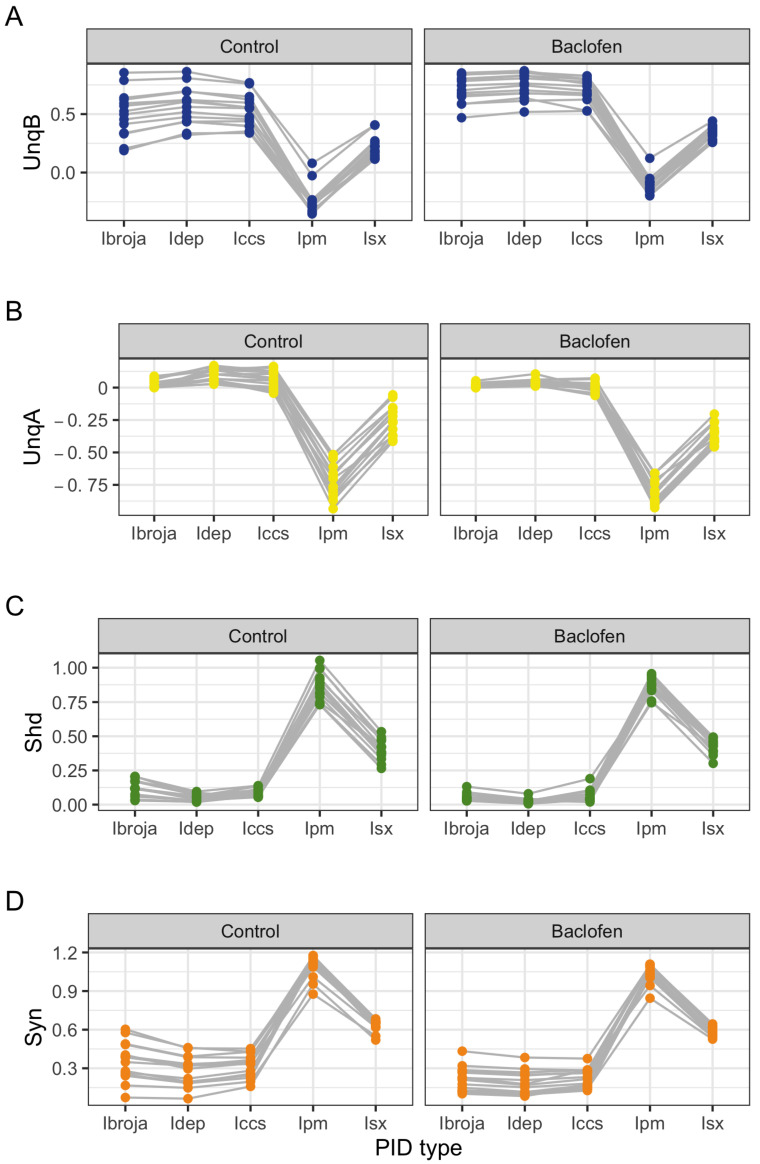
Physiological L5b neuronal recording data. Plots of each PID component, connected by each neuron, for the five PID methods: (**A**) UnqB, (**B**) UnqA, (**C**) Shd and (**D**) Syn. For each neuron under each experimental condition, the values of the PID components are given as proportions of the respective joint mutual information.

The components of the five PIDs are plotted in Figure 4. For each PID component, the values given by the Ibroja, Idep and Iccs appear to be reasonably similar for each of the Control and Baclofen conditions, although the Iccs method provides a small negative value of unique information due to the apical input for a few neurons. By way of contrast, the Ipm and Isx provide different ranges of values for each PID component, particularly evident in the plots of shared information and synergy where their ranges of values do not overlap at all with those of the PIDs Ibroja, Idep and Iccs, and they give much larger values for these components. In particular, both of the methods Ipm and Isx give very negative values of the unique information due to the apical input, much more strongly negative in the case of Ipm. Ipm also gives negative values in most cases for the unique basal information; for Isx this happens only in one case. Furthermore, the ranges of values provided by Ipm and Isx do not overlap at all except for the case of the unique information due to the basal input. The Ipm method also has values for synergy that are greater than 1, which suggests somewhat counter-intuitively that more information is transmitted in the form of synergy than is available in the joint mutual information.

Summary statistics for the sample of fifteen neurons are given in Table 2. For each of the PID components, the median values for the Ipm and Isx PIDs are very different from those produced by the Ibroja, Idep and Iccs methods, being much smaller (or negative) for UnqB and UnqA and also much larger for the components Shd and Syn. On average, The Idep and Iccs PIDs generally have slightly larger values of the unique informations than does the Ibroja PID, and correspondingly lower values for Shd and Syn. It is clear from Table 2 and Figure 4 that, for these data sets, the Ipm and Isx methods produce remarkably different PIDs. If one were interested in estimating the actual value of each component, then researchers using different methods would obtain very different values, including much larger estimates of shared information and synergy.

If the interest in the components were relative, however, and involved comparing PID components under different conditions, then perhaps the dramatic differences between the Ipm and Isx methods and the other methods would be somewhat attenuated, thus rendering the results produced by the different PID methods to be fairly similar in a relative sense, if not at an absolute level.

The purpose of the the study by Schulz et al. [28] was to examine the effect of the GABA ${}_{B}$ receptor-mediated dendritic inhibition on dendritic integration, and, in particular, whether it was associated with a change in synergy. This involves the examination of within-neuron differences of the synergy component, and means that the comparison of the relative values of synergy in the absence and presence of local baclofen application that activated GABA ${}_{B}$ receptors in the distal apical dendrite. We now turn attention to these comparisons.

#### 3.1.3. Analysis of Within-Neuron Differences in PID Components

**Figure 5:**
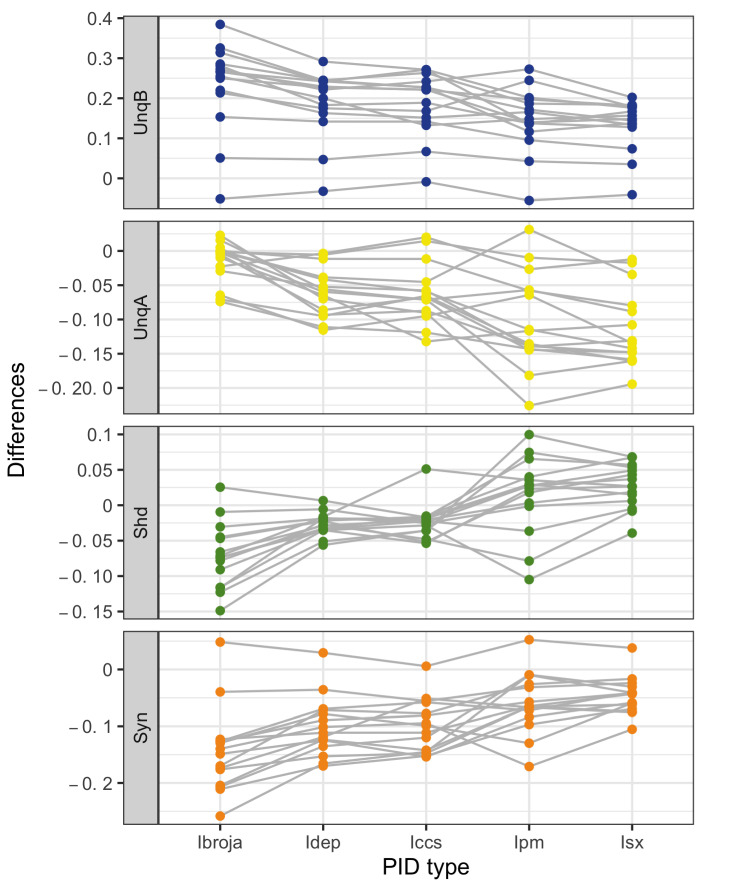
Physiological L5b neuronal recording data. Within-neuron differences in each PID component for 15 neurons, taken as Baclofen minus Control. Different vertical scales are employed for each component.

In Figure 5, the within-neuron differences in each PID component are plotted for each neuron. It is clear that the five PID methods produce differences which lie on the same scale. For UnqB, we see that the differences for all but one neuron are positive for all of the five PID methods, suggesting a general increase in UnqB in the presence of baclofen. For UnqA, most of the differences are negative for all five PIDs, thus indicating a general decrease in UnqA in the presence of baclofen. The Shd component differences reveal a possible divergence of the PID methods. Meanwhile, the Ibroja, Idep and Iccs differences are almost all negative and those for Ipm and Isx are mostly positive, thus suggesting a general increase rather than a general decrease. Apart from one neuron, the synergy differences are all negative, suggesting a general decrease in synergy in the presence of baclofen.

These observations on the plots of the component differences are also reflected in the summary statistics provided in Table 3.

#### 3.1.4. Statistical Significance

For the physiological L5b neuronal recording data [28], there is interest mainly in the synergy components. Suppose that five different researchers were to each use a different PID method and then apply a statistical test of the null hypothesis that the median value of synergy is the same in the absence and in the presence of baclofen. It turns out that all five researchers would find a significant reduction, on average, in the synergy component (all *p*-values are less than 0.001) when baclofen is present. Therefore, despite the dramatic differences between the PID results, both in the absence and presence of baclofen, all five researchers would arrive at the same formal statistical conclusion.

Suppose, however, that interest lay in the shared information component. For this component, all five PIDs do not produce the same formal statistical conclusion. The researchers using Ibroja (P < 0.001), Idep (P < 0.001) and Iccs (P < 0.006), would all find a statistically significant reduction, on average, in the shared information in the presence of baclofen. On the other hand, the researcher using Ipm would declare that there is no statistically significant difference, on average, in this component when baclofen is introduced (P = 0.2), and with Isx the researcher would declare a statistically significant *increase*, on average, in the shared component (P < 0.006).

#### 3.1.5. Unique Information Asymmetry

**Figure 6:**
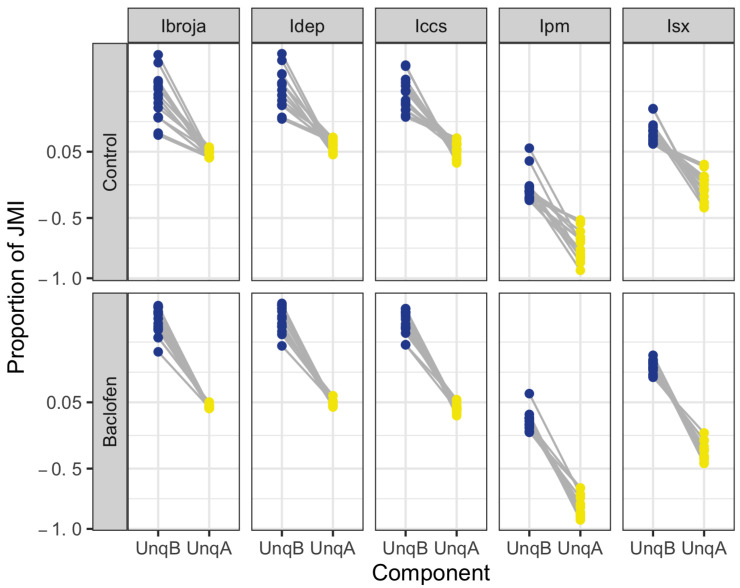
Physiological L5b neuronal recording data. A plot of the unique information asymmetries provided by each of the five PIDs for each experimental condition for 15 neurons.

Recall from Section 2.3 that the unique information asymmetry (UIA) is defined as UnqB − UnqA. For a given probability distribution, the UIA has the same value for every PID. Figure 6 shows the UnqB and UnqA values for each experimental condition and each PID. The 15 neurons have positive values of the UIA under each experimental condition. Despite the fact that the Iccs PID has a few very small negative values for UnqA, it appears that the three PIDs, Ibroja, Idep and Iccs, have very similar patterns to each other under each of the experimental conditions. On the other hand, Ipm and Isx express the asymmetries very differently, and even differently from each other. Apart from one of the 15 neurons, Ipm has negative values for UnqB and more negative values for UnqA. Hence, the asymmetries are being expressed in terms of there being much more unique apical misinformation than unique basal misinformation. For each of the 15 neurons, Isx has positive values for UnqB and negative values for UnqA. Therefore, it expresses the asymmetry as a balance of unique basal information and unique apical misinformation, with the former being larger for some neurons and smaller for others.

One property of apical amplification (CSS3) in a neuron is that there is no or little unique apical information or misinformation in a PID, coupled with the requirement that synergy or mechanistic shared information, or both, are present. It is clear in Figure 6 that both Ipm and Isx mostly have large unique misinformation components and so, while they do have large values of synergy, their PIDs are generally not compatible with property CCS3. Therefore, we focus on the other three PIDs. For Ibroja, most of the neurons have small nonnegative unique apical components, as well as appreciable synergy components (see Figure 4), and so they provide some evidence of apical amplification. The Idep and Iccs PIDs tend to produce larger values for the unique information than those obtained using Ibroja, but in the Control condition, a few neurons have small unique apical components, and this is more markedly the case in the presence of baclofen. From Figure 4, we see that several of these neurons also have appreciable values for synergy. Thus, some evidence of apical amplification is given by these neurons when using the Idep and Iccs methods, but the Ibroja method provides the strongest support for apical amplification.

Some summary statistics of the UIA values and their differences, with Bonferroni-corrected *p*-values, are provided in Table 4. The UIA is significantly positive, on average, under the control condition, and also in the presence of baclofen. When baclofen is present, the UIA is significantly larger, on average, than in the control condition. For the Ibroja, Idep and Iccs PIDs, these results, taken together with Figure 5, which shows for 14 of the neurons that in the presence of baclofen there is an increase in the transmission of unique basal information, coupled with a decrease in shared information and synergy, confirm the finding in [28] that ‘GABA ${}_{B}$ R-mediated inhibition shifts the balance toward somatic control of AP output and potently decreases apical amplification’.

### 3.2. Simulated Data from a Detailed Compartmental Model

Shai et al. [37] reported simulations of an L5b model neuron that was based on a model originally fitted to data recorded from the rat somatosensory cortex by Hay et al. [38] and then adapted it to recordings from the adult mouse visual cortex by manual manipulation of dendritic calcium and I ${}_{H}$ conductance parameters. NMDA/AMPA synapses were randomly distributed across the tuft and basal dendrites ranging in number from 0 to 300, in steps of 10, in the basal dendrites and 0 to 200, in steps of 10, in the apical dendrites. While many bursts of APs were observed, information regarding their occurrence was not recorded and, thus, is unavailable in the data set (Adam Shai, *personal communication*). Hence, we work with spike counts.

**Figure 7:**
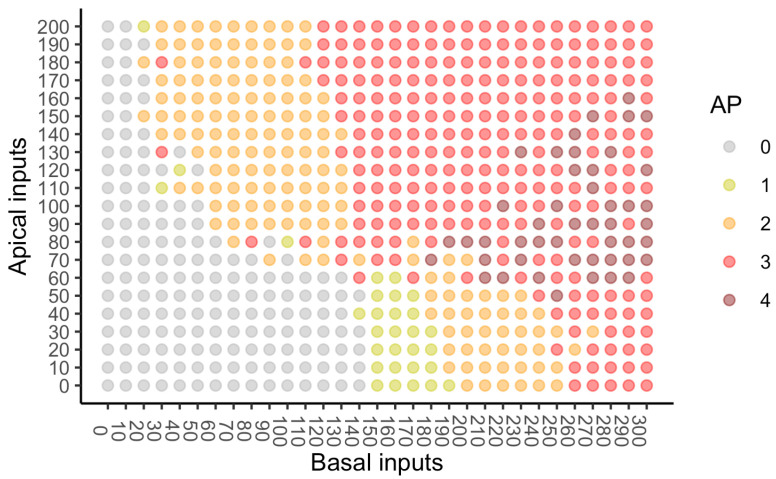
Simulated mouse L5b neuron model data. The number of action potentials emitted are provided for 651 combinations of the numbers of basal and apical inputs to the cell. The number of basal inputs ranges from 0 to 300 in steps of 10. The number of apical inputs ranges from 0 to 200 in steps of 10.

Information regarding the observed numbers of APs for the combinations of numbers of basal and apical inputs used in the study is provided in Figure 7. No APs are evoked by apical inputs when there are 0 or 10 basal inputs, but when there are no apical inputs, APs occur provided that the number of basal inputs is at least 160. This suggests that the basal input is driving when the number of apical inputs is very low. When the number of basal inputs is very low (30–50), APs occur as long as the number of apical inputs is 110 or larger, suggesting that apical inputs may be the more effective driver of AP output under certain circumstances. When the numbers of basal and apical inputs are both larger then at least 3, APs occur.

Since there are relatively few combination with 1 or 4 APs (see Figure 7), the categories of the output variable *Y* were taken to be 0, 1–2, 3–4 APs. The numbers of basal and apical inputs were not categorised. Each of the 651 observed combinations was given a probability of 1/651, with the remaining possibilities having a probability of 0, thus creating 31 values for the basal input, *B*, 21 values for the apical input, *A*, and 3 possible values for the AP count, *Y*, in a 31 by 21 by 3 probability distribution.

Several classical information measures were computed for this probability distribution (see Table 5).

In this 31 by 21 by 3 system, the joint mutual information I(Y;B,A) is 1.54 bits, while the difference I(Y;B)−I(Y;A) is 0.58 bit. Therefore, in any PID, the unique information due to basal input will be larger than that contributed by apical input by 0.58 bit. The interaction information in this system is 0.63 bit, which is 41% of the joint mutual information. Thus, without performing a PID, we can deduce, for any PID having nonnegative components, that at least 41% of the mutual information between the output *Y* and the inputs (B,A) will be due to synergy.

**Figure 8:**
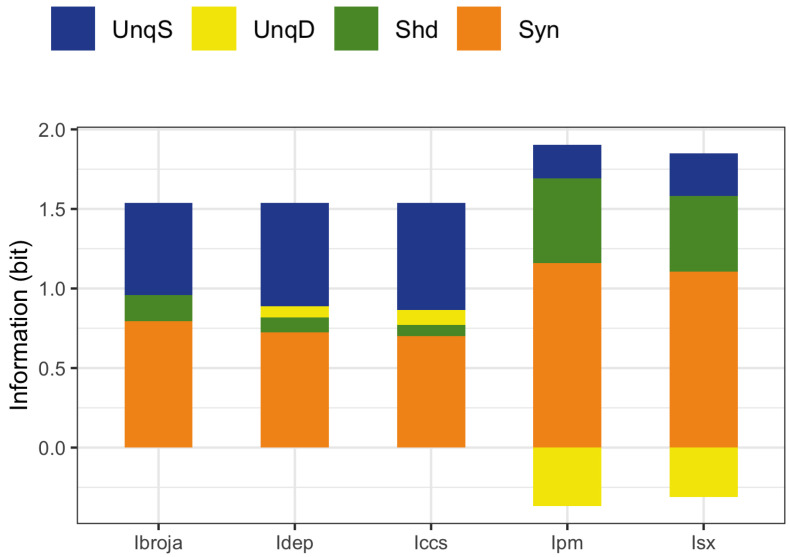
Simulated mouse L5b neuron model data. Stacked bar plots showing the values of the components for each of the five PIDs using the full data set.

To obtain the actual values of the partial information components, the five PIDs were applied to the whole data set and the results are given in Figure 8.

**Figure 9:**
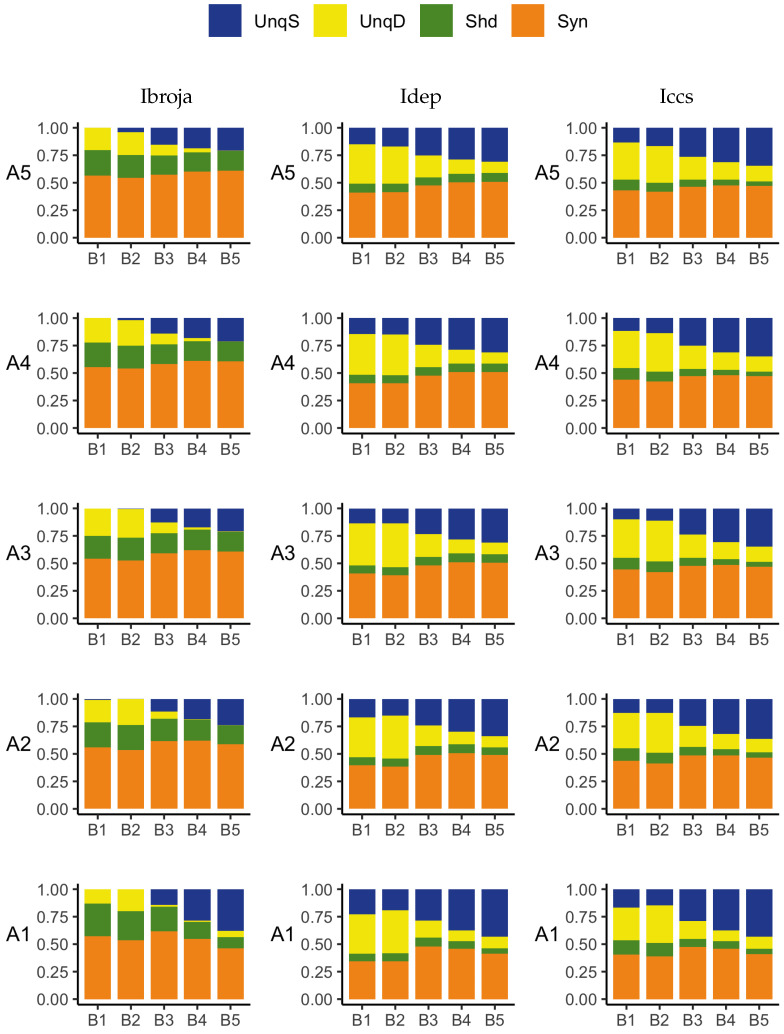
Simulated mouse L5b neuron model data. Ibroja (**left column**), Idep (**middle column**) and Iccs (**right column**) PIDs for various combinations of increasing ranges of basal inputs and increasing ranges of apical inputs: B1 (0–100), B2 (0–130), B3 (0–150), B4 (0–170), B5 (0–200) and A1 (0–100), A2 (0–130), A3 (0–150), A4 (0–170), A5 (0–200).

**Figure 10:**
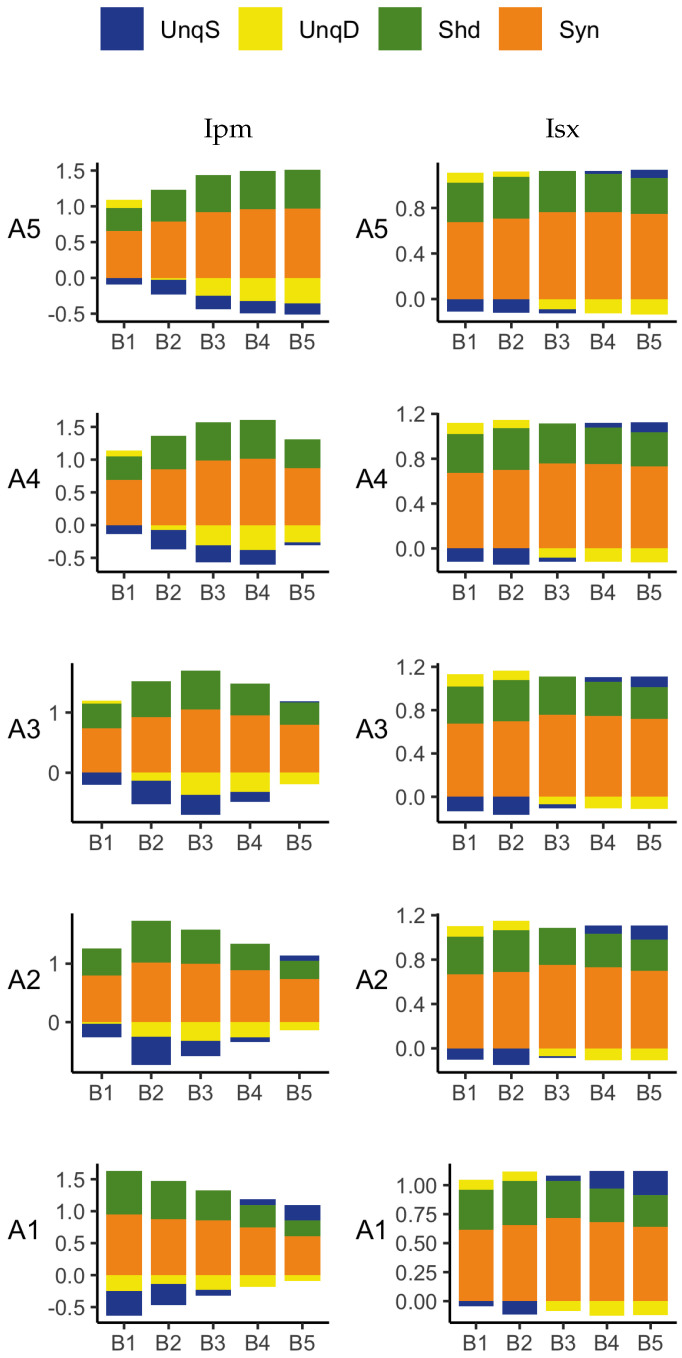
Simulated mouse L5b neuron model data. Ipm (**left column**) and Isx (**right column**) PIDs for various combinations of increasing ranges of basal inputs and increasing ranges of apical inputs: B1 (0–100), B2 (0–130), B3 (0–150), B4 (0–170), B5 (0–200) and A1 (0–100), A2 (0–130), A3 (0–150), A4 (0–170), A5 (0–200).

We now describe the bar plots in terms of percentages of the joint mutual information. As expected, all five PIDs reveal the asymmetry between the unique basal and apical components, possibly due to the disparity in numbers of apical and basal inputs—but see Figure 9 and Figure 10, which contain a combination where both basal and apical have the same range of inputs: 0–200. There are differences in how this asymmetry is expressed. The Ibroja PID has 37.7% of the joint mutual information transmitted as information unique to the basal input and no unique information due to the apical input. For the Idep PID, the respective numbers are 42.2% and 4.5%, while for the Iccs PID, they are 43.7% and 6.1%. This suggests that for all three PIDs, the basal input is primarily driving, while the apical input is mostly amplifying. Thus, these three PIDs express the asymmetry in a similar manner, with Ibroja providing the strongest suggestion of apical amplification.

On the other hand, the PIDs Ipm and Isx express the asymmetry rather differently. For Ipm, 13.8% is transmitted as information unique to the basal input, whereas 23.9% is transmitted as unique apical misinformation. The corresponding numbers for Isx are 17.4% and 20.3%. These two PIDs express the asymmetry in a similar manner. The numbers suggest that both the basal and apical inputs are driving, with the basal input transmitting information while the apical input is contributing a larger percentage as misinformation. For all five PIDs, a large percentage of the joint mutual information is transmitted as synergy, with much larger percentages for the PIDs Ipm and Isx than for the other three PIDs. Furthermore, the percentage of information transmitted as shared information is much larger for the PIDs Ipm and Isx.

#### 3.2.1. PID Analysis for Varying Strengths of Basal and Apical Input

In previous work on cooperative context-sensitivity [42,43], by utilising pre-defined probability models and particular transfer functions, it was possible to explore ideal properties, as defined in Section 2. In order to investigate such matters here with realistic data, we consider increasing subsets of numbers of basal and apical inputs, from 0–100 to 0–200 for each of the basal and apical inputs. We think of a range that has larger numbers of inputs as being stronger than a range with a smaller number of inputs. Therefore, the range 0–130 is viewed as being a stronger input than the range 0–100, and if the ranges of basal and apical inputs are both, say, 0–150, we consider the strengths of the basal and apical inputs to be equal.

We take the large range 0–100 as a baseline, as there is no information in several smaller ranges since there are no APs (Figure 7). Starting with the range 0–100, an additional 10 units were added incrementally until the range 0–200 was reached. Therefore, when the basal and apical both have the range 0–100, we see from Figure 7, that there are 11 distinct basal inputs and 11 distinct apical inputs, and for each of the 121 combinations, there are three possible values for the output. Therefore, the PIDs are based on an 11 by 11 by 3 probability distribution, with each observed combination having an equal probability of 1/121, with the remaining combinations having probability zero. Similarly, when the apical range is 0–100 and the basal range is 0–200, the PIDs are based on a 21 by 11 by 3 probability distribution, with each observed combination having equal probability 1/231, with the rest having probability zero. When the ranges are both 0–200, the PIDs are based on a 21 by 21 by 3 probability distribution with each observed cell having probability 1/441, with the rest having probability zero. Thus, there are 121 different combinations of ranges of basal and apical input. Given the different sizes of the probability distributions and the resulting differences in the values of the joint mutual information, the components of a PID in each combination were normalised by dividing by the joint mutual information for that combination. A representative sample of the 121 PIDs for each of the five methods is displayed in Figure 9 and Figure 10.

Focusing on the Ibroja results in Figure 9, we notice that there is a large synergy component in each combination, as well as an appreciable level of shared information. These levels of synergy and shared information appear to be fairly constant in all combinations for which the apical range is 0–130 or greater.

When the apical input is 0–100, however, we do notice some changes in the shared information and the synergy. As the basal range increases, there is an increase in both of these components until the range 0–150 and, thereafter, a small decrease in both as the basal range increases.

We now comment on the changes in the unique information and their relative values for these PIDs. When the basal input is 0–130 or lower, this asymmetry is negative for the subsets and there is very little unique basal information at all. On the other hand, when the basal input is 0–170 or greater, the asymmetry is positive and there is very little unique apical information. The negative asymmetry is present because the number of basal inputs is not sufficient to drive APs, whereas apical inputs are more effective at driving APs. With regard to the the positive asymmetry, now the situation changes, because basal inputs do drive APs; however, they do this in a more graded fashion than apical inputs (see Figure 7).

When the basal input is 0–150 we find that the asymmetry becomes positive, and one could say that by considering all the basal input ranges, there is a unique information asymmetry bifurcation that happens when the number of basal inputs increases from 140 to 150, irrespective of the strength of the apical input. Thus, these results reveal a much more diverse picture than the PID analysis of the whole dataset. For basal input up to 0–140, we say that the apical input is driving, whereas this begins to reverse at 0–150 and more strongly so for the larger basal input ranges. It is interesting that these patterns of results are not obtained if one were to reverse the roles of basal and apical and consider increasing apical strength; this reveals a fundamental asymmetry within the distributions.

A unique information asymmetry can also be seen when the basal and apical ranges are equal. For low values of both basal and apical, there is apical drive and this changes to basal drive when the numbers of basal and apical inputs *both* change from 140 to 150. Even though the basal and apical strengths are equal, we find that the basal input comes to dominate in terms of unique information, as the common strength increases.

These revelations also apply to the results obtained using the Idep and Iccs PIDs, although they both tend to produce larger values for the unique information components.

The Ipm and Isx results are given in Figure 10. The comments regarding the unique information asymmetry bifurcation hold also for these PIDs due to the fact that unique information asymmetry is the same for all PIDs. It is expressed very differently, however. With Ipm, both unique informations are generally negative, so the asymmetry is described as the presence of more unique basal misinformation switching to more unique apical misinformation. The Isx PID generally expresses the bifurcation in UIA as a mixture of unique apical information and unique basal misinformation changing to a mixture of unique basal information and unique apical misinformation.

#### 3.2.2. Cooperative Context-Sensitivity as Revealed by PID Analyses

In these experiments, we add further basal ranges to the previous increasing ranges of basal inputs considered in Section 3.2.1, up to 0–300. Now, we consider three fixed apical ranges with a view to assessing the effect of different fixed strengths of apical input on the basal distributions of the PID components. From Figure 7, we see that for apical ranges 110–150 and 160–200, there are five distinct input values, and so the probability distributions range from 11 by 5 by 3 (for basal 0–100), with equal probability 1/55, to 31 by 5 by 3 (for basal 0–300), with equal probability 1/155 for the observed combinations and zero for the rest. The PID components for each combination of input ranges are normalised by the joint mutual information for that combination.

**Figure 11:**
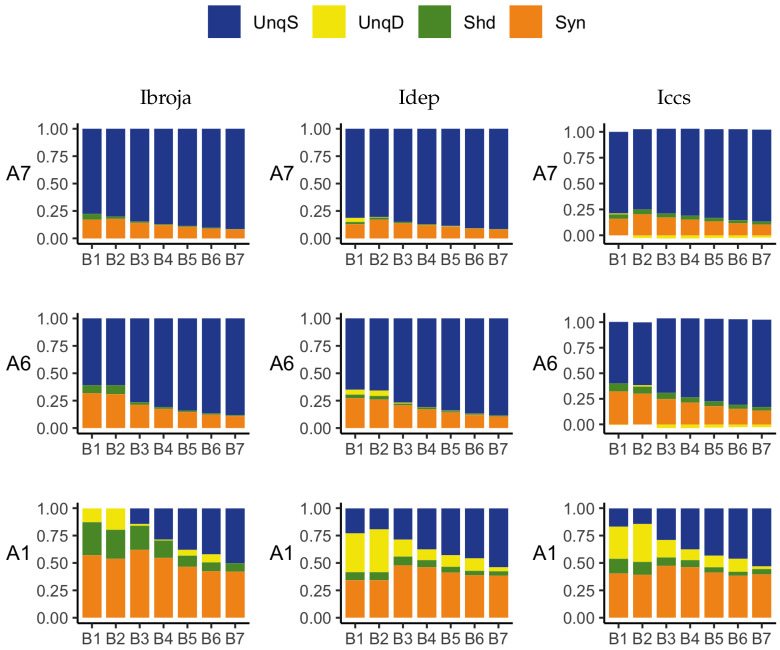
Simulated mouse L5b neuron model data. Ibroja (**left column**), Idep (**middle column**) and Iccs (**right column**) PIDs for various combinations of increasing ranges of basal inputs and three fixed ranges of apical inputs: B1 (0–100), B2 (0–130), B3 (0–150), B4 (0–170), B5 (0–200), B6 (0–250), B7 (0–300) and A1 (0–100), A6 (110–150) and A7 (160–200).

**Figure 12:**
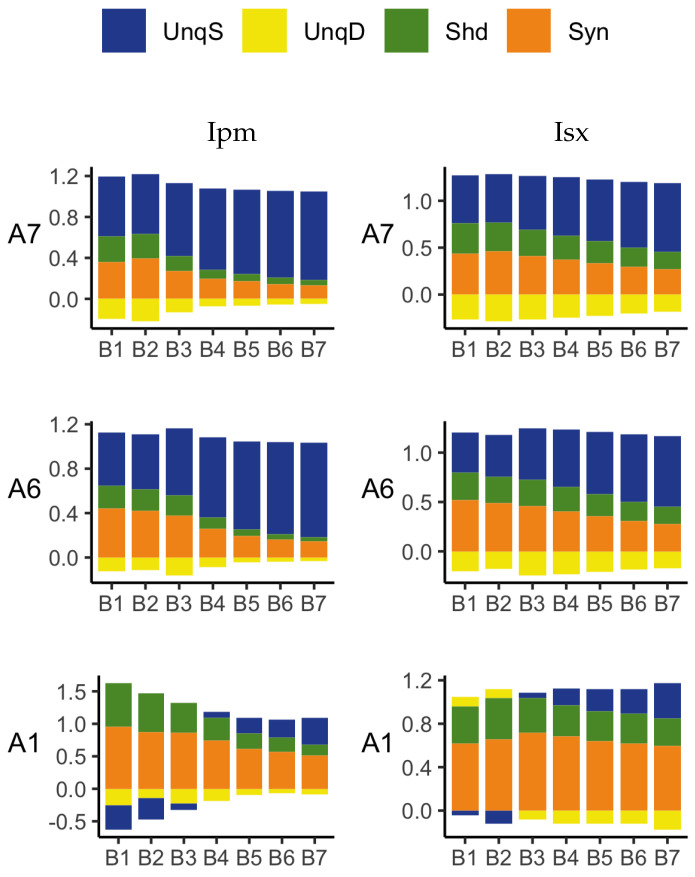
Simulated mouse L5b neuron model data. Ipm (**left column**) and Isx (**right column**) PIDs for various combinations of increasing ranges of basal inputs and three fixed ranges of apical inputs: B1 (0–100), B2 (0–130), B3 (0–150), B4 (0–170), B5 (0–200), B6 (0–250), B7 (0–300) and A1 (0–100), A6 (110–150) and A7 (160–200).

The results obtained with the Ibroja, Idep and Iccs PIDs are given in Figure 11, and those for the Ipm and Isx PIDs are in Figure 12. We now discuss the plots in Figure 7, Figure 11 and Figure 12 with regard to the ideal properties of cooperative context-sensitivity defined in Section 2, with the basal input as the ‘drive’ and the apical input as the ‘context’.

#### Properties CSS1 and CSS2

In Figure 7, we find, in the absence of apical input, that APs are emitted when the number of basal inputs is at least 150. This shows that the basal input is sufficient for the output to transmit information about the input in the absence of context. Thus, property CSS1 holds: *B* is sufficient and *A* is not necessary. When there is no basal input, we see that no APs are emitted. Thus, the apical input is not sufficient for information transmission and the basal input is necessary, and therefore, property CSS2 holds.

#### Property CSS3

In Figure 11, when the apical inputs are in ranges A6 and A7, and the basal inputs are in ranges B1–B7, the Ibroja, Idep and Iccs PIDs have large unique basal components as well as zero or small unique apical components, and synergy and some shared information are present; hence, these PIDs are consistent with property CSS3. When the apical inputs range from 0–100, only the Ibroja PIDs for 0–150, 0–170 and 0–300 basal inputs are consistent with CSS3, while for the Idep and Iccs PIDs, this is so only when the basal input range is 0–300. In Figure 12, the Ipm PID satisfies property CSS3, mainly when the basal input ranges are 0–200, 0–250 and 0–300, for all three ranges of apical input. The Isx PID does not have small components for unique apical information or misinformation and, hence, it does not produce results that are consistent with property CSS3. Therefore, property CSS3 holds most widely for the Ibroja PID, less so for Idep and Iccs, for several basal-apical combinations with the Ipm PID and not at all for Isx.

#### Property CSS4

In each of the probability distributions considered, which are defined in terms of combinations of ranges of basal and apical inputs, *B* and *A* are marginally independent, and so the source shared information, ShdS, is equal to zero. Therefore, the shared information components describe mechanistic shared information. For Ibroja, in Figure 11, when the apical input range is 0–100, and the strength of the basal input increases from 0–130 to 0–150, we see that the combined value of the UnqB, Shd and Syn information component increases, thus increasing the transmission of information about the basal input.

As the strength of the basal input is further increased, we find that the shared and synergistic components generally decrease. This provides support for property CSS4. These observations hold also for the Idep PID components as well as the Iccs components. In Figure 12, the Ipm PID does not show the increase in the combined value of the information components Shd and Syn (UnqB is misinformation), as the basal strength is increased from 0–130 to 0–150. As the strength of the basal input is increased, we do find, however, the same pattern of decreasing synergy and shared information as given by Ibroja, Idep and Iccs. Hence, Ipm is partially consistent with property CCS4. The Isx PIDs show the same characteristics that were shown with Ibroja, Idep and Iccs and so it is consistent with property CCS4.

## 4. Conclusions and Discussion

### 4.1. Rat Somatosensory Cortical L5b Pyramidal Neuron Recording Data

The PID analyses reveal that the Ibroja, Idep and Iccs methods produce broadly similar PIDs for the 15 neurons under each experimental condition, whereas the Ipm and Isx methods produce components that have very different values than those given by the Ibroja, Idep and Iccs methods. In particular, the Ipm and Isx methods produce much larger estimates of shared information and synergy. The Ipm method even produces some values for synergy that are larger than the joint mutual information, which seems nonsensical.

When the relative values of the PID components are considered—as with the within-neuron differences in the PID components used in the investigation of the effect of baclofen—these differences can be considered on the same scale for all five PID methods, and the results are generally more similar, although not for the shared information component. Statistical testing shows that for the synergy component, five independent researchers, each using one of the five methods, would arrive at the same formal statistical conclusion. Were they to consider the shared information, however, the researchers using the Ipm or Isx methods would reach a different formal statistical conclusion than obtained by those using the Ibroja, Idep and Iccs methods.

While the values of the unique information asymmetry are the same for all five methods, the asymmetry is expressed in different ways. The Ibroja, Idep and Iccs methods all exhibit strong basal drive and there is evidence of apical amplification for several neurons. Examination of the within-neuron differences and statistical testing conducted on the asymmetry values provide support for the conclusion in [28] regarding the effect of GABA ${}_{B}$ R-mediated inhibition. Neither conclusion applies to the Ipm and Isx methods.

As to the question of which method(s) to rely on, it seems wise, for probability distributions of the type considered in this study, to employ the Ibroja, Idep and Iccs methods since they give broadly similar results, rather than the Ipm or Isx method.

### 4.2. Simulated Mouse L5b Neuron Model Data

The PID analyses of the full dataset again reveal differences among the five methods, with the Ibroja, Idep and Iccs decompositions again being broadly similar. The Ipm and Isx methods transmit higher percentages of the information as synergy and shared information and an appreciable percentage as unique apical misinformation that is larger in magnitude than the transmitted unique basal information.

A richer picture emerges when various subsets of the data are analysed. When the basal and apical inputs are treated on an equal footing, and various combinations of strengths of basal and apical inputs considered, we find that there is a bifurcation in unique information asymmetry for all PIDs. While the values of the asymmetries are fixed by classical measures of mutual information, the nature of the asymmetries is only revealed by the PIDs. For Ibroja, Idep and Iccs, we find that as the strength of the basal input increases, to the extent that it is sufficient to drive AP output, there is a switch from apical drive to basal drive, and that this occurs at the same strength of basal input for every strength of apical input. We also find that this bifurcation happens when we consider combinations where the basal and apical strengths are equal. The Ipm and Isx PIDs express the asymmetry in terms of combinations of basal and apical misinformation or a combination of a unique information with a unique misinformation.

In a second exploration of subsets, increasing basal strengths were considered for three fixed apical strengths. With regard to cooperative context-sensitivity, we find that all five PIDs provide at least some support for the ideal properties. The Ibroja PID satisfies the properties to the fullest extent, with Idep and Iccs close behind. Ipm and Isx provide partial support.

A challenge in interpreting the results of any PID analysis is that the underlying reality of any system under investigation is not known; it seems there is no way to know what the true levels of the PID components actually are. It is possible to define fairly simple probability models in which there is a clear expectation that synergy, unique information, shared information, or a combination of these components, should be present, and such distributions are often used when evaluating a new PID or performing comparisons among several existing ones. For some of these standard distributions, the existing methods can agree, but on others they do not. It is, therefore, useful to consider several different PIDs and to place more emphasis on those findings where the different PIDs produce the expected results on simple probability distributions and where they produce similar results for the system under investigation. The findings obtained in the analyses considered herein with Ibroja, Iccs and Idep are very plausible since they generally accord with expectations based on the current understanding of L5b pyramidal cells.

### 4.3. Biological Implications

Twentieth century psychology and systems neuroscience were built on the assumption that neurons, in general, operate as point processors that nearly linearly sum all their synaptic inputs and signal the extent to which that net sum exceeds a threshold. Direct physiological studies of communication between the apical integration zone and the soma in layer 5 pyramidal cells of the neocortex show that assumption to be false [31,35,55,56,57]. Synaptic activation of the apical integration zone that has a limited effect on axonal AP output by itself can greatly increase the response AP output induced by more proximal synaptic inputs occurring at about the same time. This study provides the first systematic comparison of the most widely used information decomposition methods on physiological data from L5b pyramidal cells, which are known to have particularly prominent dendritic non-linearities. These analyses strongly support two important interpretations of physiological data in previous reports, despite certain limitations of the used data sets. A technical limitation of the first study is that direct current injection was used as an experimental approximation of synaptic inputs. In the second study, the model neuron is expected to provide limited accuracy in the precise AP number evoked by synaptic inputs due to the intrinsic difficulties in appropriately modeling the fast underlying conductances [37]. Despite these different constraints, our analyses converge at the conclusion that apical dendritic inputs may mainly contribute to synergy, i.e., have a modulatory role, rather than driving output information. The reason for this is that, in the investigated pyramidal neurons, apical dendritic inputs are bound to recruit an amplifying Ca${}^{2+}$ spike mechanism in the apical dendrite associated with bursts of several APs if they were to activate somatic APs directly. Therefore, apical dendritic inputs cannot provide the graded impact on AP output that basal dendritic inputs do. We can conclude that under these circumstances, the role of apical dendritic inputs is largely restricted to amplifying output rather than driving output information. Second, the results directly show that this amplification is reduced by inhibitory input to the apical zone, which implies that amplification is tightly regulated by apical inhibition. Based on recent physiological studies [58,59,60], other neuromodulatory systems are expected to play similar regulatory roles. Together, these observations support the idea that apical amplification may be an important mechanism for contextual modulation and conscious perception.

## Figures and Tables

**Table 1:** Physiological L5b neuronal recording data. Summary statistics for the sample of 15 neurons. Summary statistics of the normalised information measures, showing for each measure the median and interquartile range for the sample of 15 neurons under both experimental conditions, given as percentages of the joint mutual information.

Condition	I(Y;B)	I(Y;A)	I(Y;B|A)	I(Y;A|B)	II(Y;B;A)
Control	61.2	16.3	83.7	38.8	26.9
	(52.1, 69.8)	(11.8, 19.5)	(80.5, 88.2)	(30.2, 47.9)	(14.5, 30.0)
Baclofen	77.6	5.2	94.8	22.4	13.6
	(71.6, 86.3)	(4.3, 8.2)	(91.8, 95.7)	(13.7, 28.4)	(9.6, 22.3)

**Table 2:** Physiological L5b neuronal recording data. Summary statistics for 15 neurons. PID components are shown for each PID method and each experimental condition. The sample median (Md) and the sample quartiles (q${}_{L}$, q${}_{U}$) are stated as percentages of the joint mutual information.

		Control					Baclofen				
		**Ibroja**	**Idep**	**Iccs**	**Ipm**	**Isx**	**Ibroja**	**Idep**	**Iccs**	**Ipm**	**Isx**
	q${}_{L}$	33.7	43.5	39.7	−33.1	13.9	65.7	65.8	64.3	−16.0	13.6
UnqB	Md	49.7	51.9	47.7	−27.8	18.5	74.5	74.6	70.0	−12.7	19.2
	q${}_{U}$	60.8	65.7	61.1	−24.7	28.3	82.0	84.3	78.4	−8.2	24.0
	q${}_{L}$	0.2	6.1	1.7	−79.8	−33.3	0.0	1.9	−0.7	−88.5	−34.5
UnqA	Md	0.7	10.3	6.7	−67.8	−24.2	0.7	4.0	−0.1	−81.7	−22.2
	q${}_{U}$	5.3	13.6	12.2	−59.2	−15.6	1.9	5.5	2.1	−72.7	−15.5
	q${}_{L}$	9.4	3.9	6.3	80.0	36.7	3.5	1.8	3.6	85.5	38.8
Shd	Md	11.9	5.4	7.4	86.6	38.3	5.0	2.7	4.7	88.4	42.9
	q${}_{U}$	17.1	6.6	10.1	91.9	43.5	5.5	3.4	7.6	94.2	46.0
	q${}_{L}$	25.9	20.3	26.9	109.3	62.3	12.7	11.3	16.7	101.4	55.8
Syn	Md	38.6	32.2	34.3	112.4	65.1	21.1	17.7	23.2	106.3	58.7
	q${}_{U}$	44.4	38.6	39.2	116.6	66.2	27.2	25.9	28.5	110.0	62.3

**Table 3:** Physiological L5b neuronal recording data. Summary statistics of within-neuron differences for 15 neurons. The sample median (Md) and the sample quartiles (q${}_{L}$, q${}_{U}$) of the differences (taken as Baclofen minus Control) are provided for each PID component.

		Ibroja	Idep	Iccs	Ipm	Isx
	q${}_{L}$	21.7	16.9	14.6	12.6	13.1
UnqB	Md	26.6	22.1	22.1	14.8	14.8
	q${}_{U}$	24.2	24.2	23.4	19.2	17.8
	q${}_{L}$	−0.0	−9.0	−8.9	−14.1	−15.3
UnqA	Md	−0.0	−6.2	−6.8	−11.6	−13.5
	q${}_{U}$	0.0	−4.0	−5.1	−5.7	−8.4
	q${}_{L}$	−10.3	−3.4	−3.6	0.1	1.1
Shd	Md	−7.4	−2.5	−2.4	2.5	2.7
	q${}_{U}$	−4.6	−1.8	−1.8	3.8	5.1
	q${}_{L}$	−19.0	−13.0	−14.4	−8.0	−6.6
Syn	Md	−14.9	−10.6	−9.9	−6.5	−4.3
	q${}_{U}$	−12.6	−6.8	−5.7	−2.9	−3.6

**Table 4:** Physiological L5b neuronal recording data. Summary statistics of the values of unique information asymmetry for 15 neurons. The sample median (Md) and the sample quartiles (q${}_{L}$, q${}_{U}$) are stated as percentages of the joint mutual information. The differences between the unique information asymmetries are taken as Baclofen minus Control. The quoted *p*-values have been Bonferroni-corrected since three simultaneous tests have been performed.

	Control	Baclofen	Difference
q${}_{L}$	33.3	64.7	21.8
Md	44.6	74.5	27.9
q${}_{U}$	60.7	80.1	33.4
P	<0.0002	<0.0002	<0.0004

**Table 5:** Simulated mouse L5b neuron model data. Estimated mutual information measures. Some estimated classical mutual information measures (the unit is bit), given to two decimal places. The numbers in parentheses are the values of the measures as a percentage of the joint mutual information.

I(Y;B)	I(Y;A)	I(Y;B|A)	I(Y;A|B)	I(Y;B,A)	II(Y;B;A)	H(Y)
0.74	0.16	1.37	0.79	1.54	0.63	1.54
(48.3)	(10.7)	(89.3)	(51.7)	(100)	(41.0)	(100)

**Figure A1:**
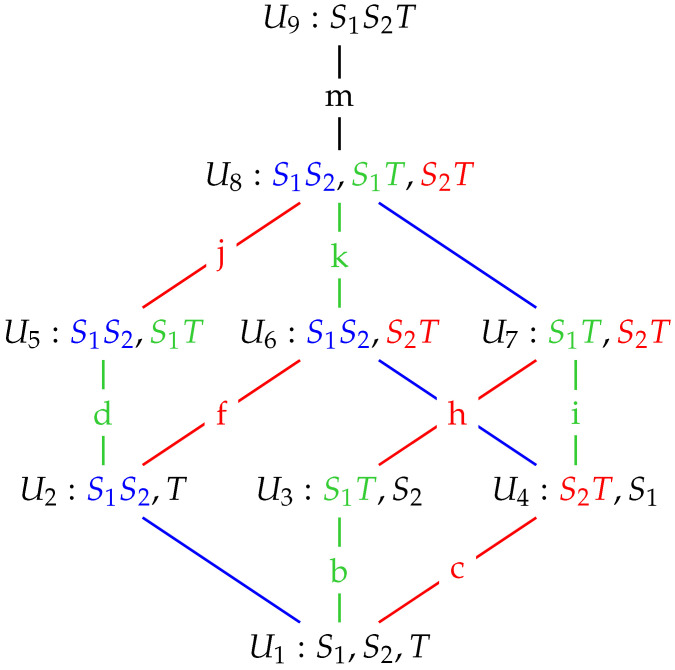
A dependency lattice of models reproduced from [63]. Edges coloured green (b, d, i, k) correspond to adding the constraint $S_{1}T$ to the model immediately below. Edges coloured red (c, f, h, j) correspond to adding the constraint $S_{2}T$ to the model immediately below.

**Figure A2:**
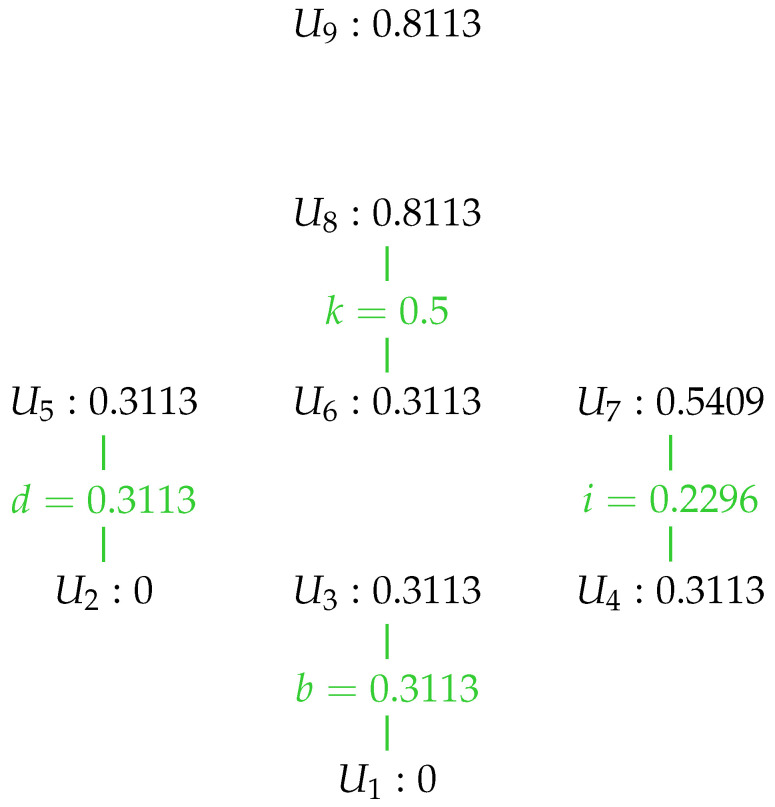
The dependency lattice with the value of the joint mutual information attached to each of the models defined in Figure A1. Edges coloured green (b, d, i, k) correspond to adding the constraint $S_{1}T$ to the model immediately below. The values of b,d,i,k are shown, and we can see that Unq$S_{1}$ = 0.2296.

**Figure A3:**
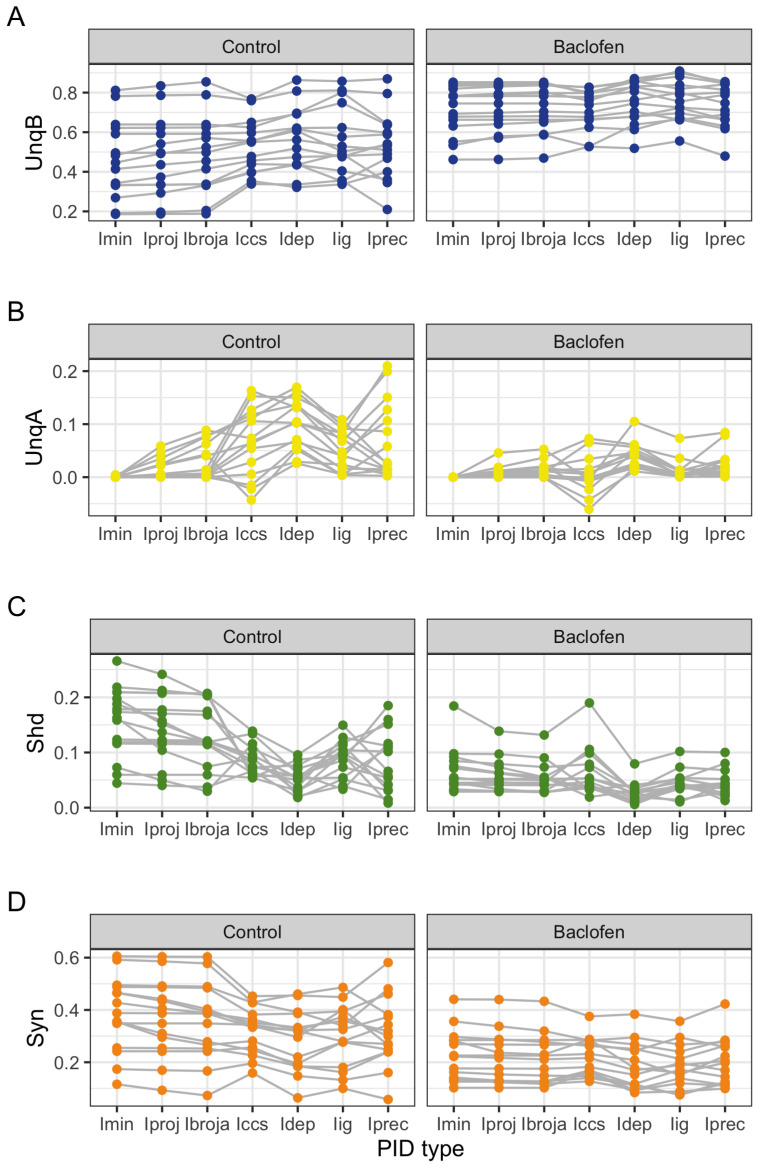
Physiological L5b neuronal recording data. Plots of each PID component, connected by each neuron, for seven PID methods: (**A**) UnqB, (**B**) UnqA, (**C**) Shd and (**D**) Syn. For each neuron under each experimental condition, the values of the PID components are given as proportions of the respective joint mutual informations.

**Figure A4:**
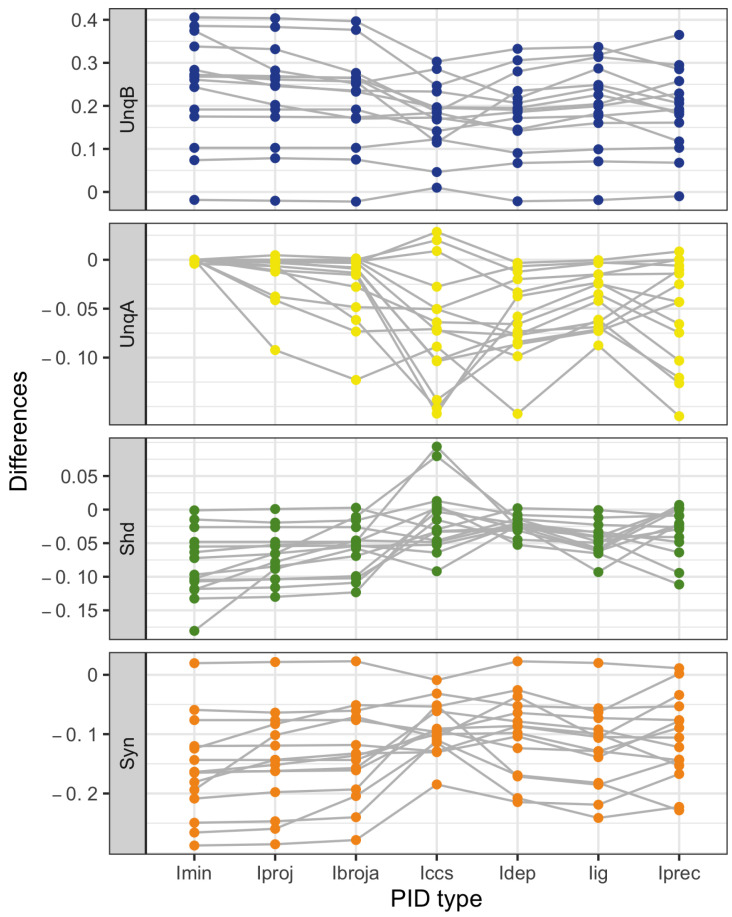
Physiological L5b neuronal recording data. Within-neuron differences in each PID component for 15 neurons, taken as Baclofen minus Control. Different vertical scales are employed for each component.

## Data Availability

For the rat somatosensory cortical L5b pyramidal neuron recording data, contact Jan Schulz. Adam Shai’s data is on ModelDB [61].

## Abbreviations

Partial Information Decomposition	
**PID**	Partial Information Decomposition, with components UnqB, UnqA, Shd and Syn, defined in Section 2.3
**Ibroja**	The PID developed by Bertschinger et al. [3]
**Iccs**	The PID developed by Ince [5]
**Idep**	The PID developed by James et al. [62]
Iig	The PID developed by Niu and Quinn [7]
**Imin**	The PID developed by Williams and Beer [1]
**Iprec**	The PID developed by Kolchinsky [10,50]
**Iproj**	The PID developed by Harder et al. [2]
**Ipm**	The PID developed by Finn and Lizier. [6]
**Isx**	The PID developed by Makkeh et al. [8]
Others	
**AMPA**	α-amino-3-hydroxy-5-methyl-4-isoxazolepropionic acid
**AP**	Action potential
**GABA**	gamma-aminobutyric acid
**GABA** ${}_{B}$	A G protein-coupled receptor for GABA
**JMI**	Joint mutual information between the output *Y* and the inputs (B,A), as defined in Section 2.2
**L5b**	Layer 5b
**NMDA**	N-methyl-D-aspartate
**UIA**	Unique information asymmetry, as defined in Section 2.4

## Appendix A. Introduction

We provide simple descriptions of the Imin, Ibroja, Idep, Iccs, Ipm and Isx partial information decompositions. The notation used varies from paper to paper but throughout this appendix we assume that there are two sources of information (inputs), $S_{1},S_{2}$, and one target (output), *T*, and that these discrete random variables have probability mass function, $p_{s_{1}s_{2}t}\equiv \mathrm{Pr}\left(S_{1}=s_{1},S_{2}=s_{2},T=t\right)$, where $s_{1},s_{2},t$ belong to the finite alphabets $A_{S_{1}},A_{S_{2}},A_{T}$, respectively. Several of the PIDs are defined for more than two sources (namely Imin, Iccs, Ipm and Isx), but here, we consider only the case of two sources. Several papers use the term *redundancy* whereas the term *shared information* will be used here.

The defining equations for a PID are as follows:

(A1) $$\begin{array}{cc} I(T;S_{1}) & =\mathrm{Shd}+\mathrm{Unq}S_{1} \end{array}$$

(A2) $$\begin{array}{cc} I(T;S_{2}) & =\mathrm{Shd}+\mathrm{Unq}S_{2} \end{array}$$

(A3) $$\begin{array}{cc} I(T;S_{1},S_{2}) & =\mathrm{Unq}S_{1}+\mathrm{Unq}S_{2}+\mathrm{Shd}+\mathrm{Syn}, \end{array}$$

where the PID components are defined in Section 2.3, albeit with slightly different notation. There are three equations in four unknowns and so to determine the PID components, a method must be provided for the computation of one of the four components. Then, the remaining components can be calculated using (A1)–(A3）.

The source papers discuss the mathematical theory underlying each PID and most provide operational interpretations, but here, for simplicity, we consider just the basic definitions and calculations on a common worked example—the AND probability distribution, defined in Table A1.

**Table A1:** The AND probability distribution.

$S_{1}$	$S_{2}$	*T*	$p(s_{1},s_{2},t)$
0	0	0	$\frac{1}{4}$
0	1	0	$\frac{1}{4}$
1	0	0	$\frac{1}{4}$
1	1	1	$\frac{1}{4}$

We now state in Table A2 some marginal probability distributions that will be required in the calculations to follow. In all the calculations, logarithms are taken to base 2, so the unit is the *bit*.

**Table A2:** The univariate and bivariate marginal distributions of the AND probability distribution.

(a)				(b)				(c)			
$s_{1}$		0	1	$s_{2}$		0	1	*t*		0	1
$p(s_{1})$		$\frac{1}{2}$	$\frac{1}{2}$	$p(s_{2})$		$\frac{1}{2}$	$\frac{1}{2}$	p(t)		$\frac{3}{4}$	$\frac{1}{4}$
(d)				(e)				(f)			
		*t*				*t*				$s_{2}$	
	$p(s_{1},t)$	0	1		$p(s_{2},t)$	0	1		$p(s_{1},s_{2})$	0	1
$s_{1}$	0	$\frac{1}{2}$	0	$s_{2}$	0	$\frac{1}{2}$	0	$s_{1}$	0	$\frac{1}{4}$	$\frac{1}{4}$
	1	$\frac{1}{4}$	$\frac{1}{4}$		1	$\frac{1}{4}$	$\frac{1}{4}$		1	$\frac{1}{4}$	$\frac{1}{4}$

We now compute the mutual information in (A1)–(A3) that will be required later to determine the PIDs. Recall that the Shannon entropy has the form [44]:

(A4) $$H\left(X\right)=-\sum_{x\in A_{X}}p_{x}\log p_{x}$$

Using (A4), we obtain the following values for entropies and mutual information: $\begin{array}{cccc} H(S_{1}) & =-\frac{1}{2}\log\frac{1}{2}-\frac{1}{2}\log\frac{1}{2} & = & \;\;1\\ H(S_{2}) & =-\frac{1}{2}\log\frac{1}{2}-\frac{1}{2}\log\frac{1}{2} & = & \;\;1\\ H(T) & =-\frac{3}{4}\log\frac{3}{4}-\frac{1}{4}\log\frac{1}{4} & ≐ & \;\;0.8113\\ H(T,S_{1}) & =-\frac{1}{2}\log\frac{1}{2}-\frac{1}{4}\log\frac{1}{4}-\frac{1}{4}\log\frac{1}{4} & = & \;\;\frac{3}{2}\\ H(T,S_{2}) & =-\frac{1}{2}\log\frac{1}{2}-\frac{1}{4}\log\frac{1}{4}-\frac{1}{4}\log\frac{1}{4} & = & \;\;\frac{3}{2}\\ H(S_{1},S_{2}) & =4\times (-\frac{1}{4}\log\frac{1}{4}) & = & \;\;2\\ H(T,S_{1},S_{2}) & =4\times (-\frac{1}{4}\log\frac{1}{4}) & = & \;\;2 \end{array}$

(A5) $$\begin{array}{cccc} I(T;S_{1}) & =H\left(T\right)+H\left(S_{1}\right)-H\left(T,S_{1}\right) & ≐ & \;\;0.3113 \end{array}$$

(A6) $$\begin{array}{cccc} I(T;S_{2}) & =H\left(T\right)+H\left(S_{2}\right)-H\left(T,S_{2}\right) & ≐ & \;\;0.3113 \end{array}$$

(A7) $$\begin{array}{cccc} I(T;S_{1},S_{2}) & =H\left(T\right)+H\left(S_{1},S_{2}\right)-H\left(T,S_{1},S_{2}\right) & ≐ & \;\;0.8113 \end{array}$$

Equations (A1)–(A3) and (A5)–(A7) will be used in the sequel when computing the six PIDs.

## Appendix B. The Imin PID

Williams and Beer [1] formulated a measure of shared information in a semi-pointwise manner. They expressed the mutual information between a source *S* and the target, *T*, as follows:

$$\begin{array}{cc} I(T;S) & =\sum_{t\in A_{T}}\;\sum_{s\in A_{S}}p\left(s,t\right)\log\frac{p(s,t)}{p(s)p(t)}\\ \; & =\sum_{t\in A_{T}}p\left(t\right)I\left(T=t;S\right), \end{array}$$

where:

(A8) $$I\left(T=t;S\right)=\sum_{s\in A_{S}}p\left(s|t\right)i\left(t;s\right)=\frac{1}{p(t)}\sum_{s\in A_{S}}p\left(s,t\right)i\left(t;s\right)$$

is the *specific information* that a source, *S*, provides about the particular outcome T=t, and:

$$i\left(t;s\right)=\log\frac{p(t,s)}{p(t)p(s)}=\log\frac{p(t|s)}{p(t)}$$

is the local or pointwise mutual information between the particular realisations T=t and S=s. Then, the shared information is defined [1] to be the expected value of the minimum information that any source provides about each outcome of *T*:

(A9) $$I_{\min}\left(T;S_{1},S_{2}\right)\sum_{t\in A_{T}}p\left(t\right)\min\left\{I\left(T=t;S_{1}\right),I\left(T=t;S_{2}\right)\right\}.$$

### Application of Imin to the AND Distribution

Applying this to the AND probability distribution, we note from Table A2 (a,b,d,e) that $S_{1}$ and $S_{2}$ have the same distribution, and that $\left(S_{1},T\right)$ and $\left(S_{2},T\right)$ have the same distribution. Therefore, $I\left(T=t;S_{1}\right)=I\left(T=t;S_{2}\right)$ and we need only to consider $I(T=t;S_{1})$. In what follows, we use 0log0=0.

Using the fact that p(s|t)=p(s,t)/p(t) in (A8) we have that:

$$I\left(T=0;S_{1}\right)=\frac{4}{3}\left(\frac{1}{2}\log\frac{\frac{1}{2}}{\frac{1}{2}\frac{3}{4}}+\frac{1}{4}\log\frac{\frac{1}{4}}{\frac{1}{2}\frac{3}{4}}\right)=\frac{1}{3}\log\frac{32}{27}$$

and:

$$I\left(T=1;S_{1}\right)=4\times \left(0\log0+\frac{1}{4}\log\frac{\frac{1}{4}}{\frac{1}{2}\frac{1}{4}}\right)=1.$$

Therefore from (A9),

$$\mathrm{Shd}\equiv I_{\min}\left(T;S_{1},S_{2}\right)=\frac{1}{4}\left(\log\frac{32}{27}+1\right)=\frac{3}{2}-\frac{3}{4}\log3≐0.3113.$$

Using (A1)–(A3) and (A5)–(A7) we have:

$$\begin{array}{cc} \mathrm{Unq}S_{1} & =I(T;S_{1})-\mathrm{Shd}=0,\\ \mathrm{Unq}S_{2} & =I(T;S_{2})-\mathrm{Shd}=0,\\ \mathrm{Syn} & =I\left(T;S_{1},S_{2}\right)-\mathrm{Shd}\;-\mathrm{Unq}S_{1}-\mathrm{Unq}S_{2}=\frac{1}{2}. \end{array}$$

## Appendix C. The Ibroja PID

The paper by Bertschinger et al. [3] provides an optimisation approach to find each of the PID components. We consider the convex optimisation problem for the calculation of synergy. Let Δ be the set of all joint distributions for $S_{1},S_{2},T$, and denote the given distribution by *P*. Consider the class of distributions:

$$\Delta_{P}=\left\{Q\in \Delta :q\left(s_{1},t\right)=p\left(s_{1},t\right)\;\;\mathrm{and}\;\;q\left(s_{2},t\right)=p\left(s_{2},t\right),\;\;\mathrm{for}\;\mathrm{all}\;\;s_{1}\in A_{S_{1}},s_{2}\in A_{S_{2}},t\in A_{T}\right\}$$

in which the $\left(S_{1},T\right)$ and $\left(S_{2},T\right)$ marginal distributions are constrained to be equal to the corresponding marginal distributions of the given distribution *P*.

The synergy is given by [3]:

(A10) $$I\left(T;S_{1},S_{2}\right)-\min_{Q\in \Delta }I_{Q}\left(T;S_{1},S_{2}\right),$$

where the subscript *Q* means that the joint mutual information is calculated using the distribution *Q*.

### Application of Ibroja to the AND Distribution

Here the probability distribution *P* is the AND distribution. We let $q_{s_{1}s_{2}t}\equiv q\left(t,s_{1},s_{2}\right)$ be the probability mass function of the distribution *Q*. For the AND distribution, the $\left(S_{1},T\right)$ and $\left(S_{2},T\right)$ marginal distributions are given in Table A2(d,e).

Applying the marginal constraints, we have for the $\left(S_{1},T\right)$ and $\left(S_{2},T\right)$ marginal distributions, the following equations to be solved for the $q_{s_{1}s_{2}t},\left(s_{1}=0,1;s_{2}=0,1;t=0,1\right),\left(0\leq q_{s_{1}s_{2}t}\leq 1\right).$

$$\begin{array}{cccc} (s_{1}=0,t=0)\; & q_{000}+q_{010} & =\frac{1}{2}, & \;\;\;\left(s_{2}=0,t=0\right)\;q_{000}+q_{100}=\frac{1}{2} \end{array}$$

(A11) $$\begin{array}{cccc} (s_{1}=0,t=1)\; & q_{001}+q_{011} & =0, & \;\;\;\left(s_{2}=0,t=1\right)\;q_{001}+q_{101}=0\\ (s_{1}=1,t=0)\; & q_{100}+q_{110} & =\frac{1}{4}, & \;\;\;\left(s_{2}=1,t=0\right)\;q_{010}+q_{110}=\frac{1}{4} \end{array}$$

(A12) $$\begin{array}{cccc} (s_{1}=1,t=1)\; & q_{101}+q_{111} & =\frac{1}{4}, & \;\;\;\left(s_{2}=1,t=1\right)\;q_{011}+q_{111}=\frac{1}{4} \end{array}$$

From (A11), $q_{001}=q_{011}=q_{101}=0.$ Therefore, from (A12), $q_{111}=\frac{1}{4}.$

If we let $q_{000}=\alpha$, then the other $q_{s_{1}s_{2}t}$ can be expressed in terms of α, as follows:

$$q_{000}=\alpha ,\;q_{010}=\frac{1}{2}-\alpha ,\;q_{100}=\frac{1}{2}-\alpha ,\;q_{110}=\alpha -\frac{1}{4},$$

for $\frac{1}{4}\leq \alpha \leq \frac{1}{2}.$ Note that while the marginal distributions of $\left(S_{1},T\right)$ and $\left(S_{2},T\right)$ are kept fixed (as well as the univariate marginal distributions of $S_{1},S_{2}$ and *T*), the marginal distribution of $\left(S_{1},S_{2}\right)$ now depends on α and it is allowed to change during the optimisation:

$$\begin{array}{cc} \mathrm{Pr}\left(S_{1}=0,S_{2}=0\right)=\mathrm{Pr}\left(S_{1}=1,S_{2}=1\right) & =\alpha ,\\ \mathrm{Pr}\left(S_{1}=0,S_{2}=1\right)=\mathrm{Pr}\left(S_{1}=1,S_{2}=0\right) & =\frac{1}{2}-\alpha . \end{array}$$

We now find an expression for $I_{Q}\left(T;S_{1},S_{2}\right)$ in terms of α.

(A13) $$\begin{array}{cc} H_{Q}\left(T\right) & =H\left(T\right)=2-\frac{3}{4}\log3 \end{array}$$

(A14) $$\begin{array}{cc} H_{Q}\left(S_{1},S_{2}\right) & =-2\alpha \log\alpha -2\left(\frac{1}{2}-\alpha \right)\log\left(\frac{1}{2}-\alpha \right) \end{array}$$

(A15) $$\begin{array}{cc} H_{Q}\left(S_{1},S_{2},T\right) & =-\alpha \log\alpha -2\left(\frac{1}{2}-\alpha \right)\log\left(\frac{1}{2}-\alpha \right)-\left(\alpha -\frac{1}{4}\right)\log\left(\alpha -\frac{1}{4}\right)-\frac{1}{4}\log\frac{1}{4} \end{array}$$

Therefore, from (A3) we find that:

(A16) $$I_{Q}\left(T;S_{1},S_{2}\right)=H\left(T\right)-\alpha \log\alpha +\left(\alpha -\frac{1}{4}\right)\log\left(\alpha -\frac{1}{4}\right)+\frac{1}{4}\log\frac{1}{4}.$$

For $\frac{1}{4}<\alpha <\frac{1}{2},$ the first derivative of $I_{Q}\left(T;S_{1},S_{2}\right)$ is $\log\{\left(\alpha -\frac{1}{4}\right)/\alpha \}<0$. Therefore, $I_{Q}\left(T;S_{1},S_{2}\right)$ is strictly decreasing with respect to α for $\frac{1}{4}<\alpha <\frac{1}{2}$ and so the global minimum is attained when $\alpha =\frac{1}{2}.$ Using Equations (A7), (A10), (A13) and (A16), we find that Syn = $\frac{1}{2}.$

Therefore, using (A1)–(A3) and (A5)–(A7) we have:

(A17) $$\begin{array}{cc} \mathrm{Unq}S_{1} & =I\left(T;S_{1},S_{2}\right)-I\left(T;S_{2}\right)-\mathrm{Syn}=\frac{1}{2}-\frac{1}{2}=0, \end{array}$$

(A18) $$\begin{array}{cc} \mathrm{Unq}S_{2} & =I\left(T;S_{1},S_{2}\right)-I\left(T;S_{1}\right)-\mathrm{Syn}=\frac{1}{2}-\frac{1}{2}=0, \end{array}$$

(A19) $$\begin{array}{cc} \mathrm{Shd} & =I\left(T;S_{1}\right)-\mathrm{Unq}S_{1}≐0.3113. \end{array}$$

Thus, we have found the Ibroja PID.

## Appendix D. The Idep PID

In [62], a method is proposed to quantify the unique information conveyed by the source variables, $S_{1},S_{2}$, about the target, *T*. The formulation in [62] starts from a lattice of maximum entropy models that are determined by marginal constraints, where the lattice structure comes from the hierarchy of the marginal constraints; see Figure A1. The following description is based on [63]:

For example, $U_{1}$ represents the maximum entropy distribution, having probability mass function $q(s_{1},s_{2},t)$, under the constraints that the univariate marginals match exactly the univariate marginals of the given distribution, which has probability mass function $p(s_{1},s_{2},t)$. That is: $q\left(s_{1}\right)=p\left(s_{1}\right),\;q\left(s_{2}\right)=p\left(s_{2}\right),\;q\left(t\right)=p\left(t\right).$

$U_{2}$ represents the maximum entropy distribution subject to the constraints $q\left(s_{1},s_{2}\right)=p\left(s_{1},s_{2}\right),q\left(t\right)=p\left(t\right)$.

$U_{5}$ represents the maximum entropy distribution subject to the constraints $q\left(s_{1},s_{2}\right)=p\left(s_{1},s_{2}\right),q\left(s_{1},t\right)=p\left(s_{1},t\right)$, and so on.

The lattice structure arises from the higher order constraints enforcing corresponding lower order constraints, so that, for example, imposing a bivariate marginal constraint such as $q\left(s_{1},t\right)=p\left(s_{1},t\right)$ means also that the lower order constraints $q\left(s_{1}\right)=p\left(s_{1}\right)$ and q(t)=p(t) also hold.

The coloured edges correspond to adding a pairwise marginal constraint. Blue edges represent the constraint $S_{1}S_{2}$, i.e., preserving the pairwise dependency between $S_{1}$ and $S_{2}$. Green and red labelled edges correspond to the addition of the $S_{1}T$ and the $S_{2}T$ dependencies, respectively.

For each model $U_{1}\ldots U_{8}$, we calculate the mutual information between the sources, $S_{1},S_{2}$ and target, *T*, under that model: $I_{U_{i}}\left(T;S_{1},S_{2}\right)$. The unique information in $S_{1}$ is then obtained as the minimum change in $I_{U_{i}}$ along all the green edges due to the addition of the $S_{1}T$ constraint to the model below. Similarly, the unique information in $S_{2}$ can be obtained as the minimum change in $I_{U_{i}}$ along all the red edges due to the addition of the $S_{2}T$ constraint to the model below. So, for example, the edge value *i* is equal to $I_{U_{7}}-I_{U_{4}}$. It suffices to compute just one unique information [62].

The models $U_{1}-U_{8}$ are actually the well-known loglinear models used in statistical modelling [64,65] and they were fitted here using an iterative proportional fitting algorithm [66], available in base R [49]. In fact, all of the models have closed form solutions except for model $U_{8}$.

### Application Idep to the AND Distribution

Figure A2 shows the value of the joint mutual information for each of the fitted models in the lattice. We find that edge *i* gives the the minimum of the green edge values, and so Unq$S_{1}$ = 0.2296.

Therefore, using (A1)–(A3) and (A5)–(A7), we have:

$$\begin{array}{cc} \mathrm{Shd} & =I\left(T;S_{1}\right)-\mathrm{Unq}S_{1}≐0.3113-0.2296≐0.0817,\\ \mathrm{Unq}S_{2} & =I(T;S_{2})-\mathrm{Shd}≐0.3113-0.0817≐0.2296,\\ \mathrm{Syn} & =I\left(T;S_{1},S_{2}\right)-I\left(T;S_{2}\right)-\mathrm{Unq}S_{1}≐0.8113-0.3113-0.2296≐0.2704. \end{array}$$

Thus we have found the Idep PID.

## Appendix E. The Iccs PID

The Iccs method [5] is the first of the three pointwise methods to be considered. In this method, a measure of shared information is defined by considering each realisation individually in a pointwise manner. The pointwise mutual information can be considered as a *change in surprisal*, as follows.

$$i\left(t;s_{1}\right)=\log\frac{p(t,s_{1})}{p\left(t\right)p\left(s_{1}\right)}=\log\frac{1}{p(t)}-\log\frac{1}{p(t|s_{1})},$$

which is the surprisal at observing the value *t* of *T* minus the surprisal at observing T=t once it is known that $S_{1}=s_{1}$. The pointwise interaction information may be written in terms of changes in surprisal terms as:

$$\begin{array}{cc} i(t;s_{1};s_{2}) & =\log\frac{p(t|s_{1},s_{2})}{p(t)}-\log\frac{p(t|s_{1})}{p(t)}-\log\frac{p(t|s_{2})}{p(t)}\\ \; & \equiv \Delta_{t}h\left(s_{1},s_{2}\right)-\Delta_{t}h\left(s_{1}\right)-\Delta_{t}h\left(s_{2}\right). \end{array}$$

and the negation of this, the pointwise coinformation $c(t;s_{1};s_{2})$, measures the overlap in the change of surprisal about *t* between the values $s_{1}$ and $s_{2}$; see [5].

The Iccs method considers each realisation in the probability distribution and imposes the requirement that for a realisation to contribute to the shared information, the signs of

$$\Delta_{t}h\left(s_{1},s_{2}\right),\;\;\Delta_{t}h\left(s_{1}\right),\;\;\Delta_{t}h\left(s_{2}\right)\;\mathrm{and}\;c\left(t;s_{1};s_{2}\right)$$

must all be the same, either positive or negative.

### Application of Iccs to the AND Distribution

We now apply this to the AND distribution. The various terms are computed using the maximum entropy distribution, which has the same pairwise marginal distributions as the given distribution, *P*. For the given AND distribution, the maximum entropy distribution is equal to the AND distribution itself. In fact, the maximum entropy distribution is model $U_{8}$ from Figure A1 and Figure A2. Therefore, we use the marginal tables for the AND distribution that are given in Table A1 and Table A2(a–f). The results are given in Table A3. We note that logx>0 when x>1 and logx<0 when x<1.

**Table A3:** The pointwise calculations in the Iccs method applied to the AND distribution.

$\left(s_{1},s_{2},t\right)$	$\Delta_{t}h\left(s_{1}\right)$	$\Delta_{t}h\left(s_{2}\right)$	$\Delta_{t}h\left(s_{1},s_{2}\right)$	$c(t;s_{1};s_{2})$	Allowed?
(0,0,0)	$\log\frac{4}{3}$	$\log\frac{4}{3}$	$\log\frac{4}{3}$	$\log\frac{4}{3}$	Yes
(0,1,0)	$\log\frac{4}{3}$	$-\log\frac{3}{2}$	$\log\frac{4}{3}$	$\;-\log\frac{3}{2}$	No
(1,0,0)	$-\log\frac{3}{2}$	$\log\frac{4}{3}$	$\log\frac{4}{3}$	$-\log\frac{3}{2}$	No
(1,1,1)	1	1	2	0	Yes

Only the realisations (0,0,0) and (1,1,1) are allowed to contribute to the estimate of the shared information, since all of the required values have the same sign, which is positive in both cases. The other two realisations are excluded since there is a sign mismatch for each of them. For (0,1,0), $\Delta_{t}h\left(s_{1}\right)$ is positive whereas $\Delta_{t}h\left(s_{2}\right)$ is negative, and vice-versa for realisation (1,0,0).

Averaging the allowed pointwise values of the coinformation $c(t;s_{1};s_{2})$ with respect to the AND probability distribution, we find that the shared information is:

$$\mathrm{Shd}=\frac{1}{4}\left(\log\frac{4}{3}+0\right)≐0.1038.$$

Therefore, using (A1)–(A3) and (A5)–(A7), we have:

$$\begin{array}{ccc} \mathrm{Unq}S_{1} & =I(T;S_{1})-\mathrm{Shd}≐0.3113-0.1038 & ≐0.2075,\\ \mathrm{Unq}S_{2} & =I(T;S_{2})-\mathrm{Shd}≐0.3113-0.1038 & ≐0.2075,\\ \mathrm{Syn} & =I\left(T;S_{1},S_{2}\right)-I\left(T;S_{2}\right)-\mathrm{Unq}S_{1}≐0.8113-0.3113-0.2075 & ≐0.2925. \end{array}$$

Thus, we have found the Iccs PID.

## Appendix F. The Ipm PID

Finn and Lizier [6] provide a fully pointwise approach in which measures of each of the four PID components are defined for each realisation in the probability distribution. In other words, the defining equations in (A1)–(A3) are expressed in a pointwise manner. For each realisation, the pointwise shared information is expressed as the difference of two nonnegative terms, the first of which is informative and the second misinformative. The functional forms used in the following definitions were shown in [67] to be the unique functions, which satisfy four postulates that are motivated by consideration of probability mass exclusions, which we will meet when discussing the Isx method in Appendix G.

The informative part of the pointwise shared information is [6]:

(A20) $${\mathrm{Shd}}^{+}=\min\left\{h\left(s_{1}\right),h\left(s_{2}\right)\right\},\;\mathrm{with}\;h\left(s_{i}\right)=-\log p\left(s_{i}\right),\;\left(i=1,2\right).$$

The misinformative part of the pointwise shared information is defined to be [6]:

(A21) $${\mathrm{Shd}}^{-}=\min\left\{h\left(s_{1}|t\right),h\left(s_{2}|t\right)\right\},\;\mathrm{with}\;h\left(s_{i}|t\right)=-\log p\left(s_{i}|t\right).$$

The pointwise shared information for a given realisation is then defined to be [6]:

$${\mathrm{Shd}}^{p}={\mathrm{Shd}}^{+}-{\mathrm{Shd}}^{-}.$$

The average value of the shared information is obtained by taking the average of the Shd${}^{p}$ terms with respect to the AND distribution. We focus here on the average PID, but the full pointwise PID can be expressed in much more detail at the atomic level [6] and separate pointwise PIDs for the informative terms (termed specificities) and for the misinformative terms (termed ambiguities) can be computed.

### Application of the Ipm Method to the AND Distribution

We now compute the average PID for the AND distribution using the marginal probability distributions in Table A2. The required terms are given in Table A4.

**Table A4:** Pointwise calculations for application of the Ipm method to the AND distribution.

$\left(s_{1},s_{2},t\right)$	$h(s_{1})$	$h(s_{2})$	Shd${}^{+}$	$h(s_{1}|t)$	$h(s_{2}|t)$	Shd${}^{-}$	Shd${}^{p}$
(0,0,0)	1	1	1	$\log\frac{3}{2}$	$\log\frac{3}{2}$	$\log\frac{3}{2}$	$\log\frac{4}{3}$
(0,1,0)	1	1	1	$\log\frac{3}{2}$	log3	$\log\frac{3}{2}$	$\log\frac{4}{3}$
(1,0,0)	1	1	1	log3	$\log\frac{3}{2}$	$\log\frac{3}{2}$	$\log\frac{4}{3}$
(1,1,1)	1	1	1	0	0	0	1

For example, consider the (0,0,0) realisation in the first row. From Table A2(a,b), $\mathrm{Pr}(S_{1}=0)=0.5$, and so from (A20) $h(s_{1})=-\log0.5=1$. Similarly, $\mathrm{Pr}(S_{2}=0)$ = 0.5 and so from (A20), $h(s_{2})=1$. Therefore, Shd${}^{+}=\min\left(1,1\right)=1.$

Furthermore, from Table A2(c–e), we have that:

$$\mathrm{Pr}\left(S_{1}=0|T=0\right)=\mathrm{Pr}\left(S_{1}=0\;\;\&\;\;T=0\right)/\mathrm{Pr}\left(T=0\right)=\left(1/2\right)/\left(3/4\right)=3/2$$

and so, using (A21), we find that $h\left(s_{1}|t\right)=\log\frac{3}{2}.$ Similarly, $h\left(s_{2}|t\right)=\log\frac{3}{2}$ and, therefore,

$${\mathrm{Shd}}^{-}=\min\left(\log\frac{3}{2},\log\frac{3}{2}\right)=\log\frac{3}{2}.$$

The overall contribution towards the shared information for this realisation is:

$$\mathrm{Shd}={\mathrm{Shd}}^{+}-{\mathrm{Shd}}^{-}=1-\log\frac{3}{2}=\log\frac{4}{3}.$$

The other terms are computed in a similar manner.

The average of the pointwise shared information taken with respect to the AND distribution is:

$$\mathrm{Shd}=\frac{1}{4}\log\frac{4}{3}+\frac{1}{4}\log\frac{4}{3}+\frac{1}{4}\log\frac{4}{3}+\frac{1}{4}≐0.5613.$$

Therefore, using (A1)–(A3) and (A5)–(A7), we have:

$$\begin{array}{ccc} \mathrm{Unq}S_{1} & =I(T;S_{1})-\mathrm{Shd}≐0.3113-0.5613 & ≐-0.2497,\\ \mathrm{Unq}S_{2} & =I(T;S_{2})-\mathrm{Shd}≐0.3113-0.5613 & ≐-0.2497,\\ \mathrm{Syn} & =I\left(T;S_{1},S_{2}\right)-I\left(T;S_{2}\right)-\mathrm{Unq}S_{1}≐0.8113-0.3113+0.2497 & ≐0.7497. \end{array}$$

Thus, we have found the Ipm PID.

## Appendix G. The Isx PID

The Isx PID [8] is another fully pointwise PID method, which builds on the work of [6,67] and introduces a new method for computing pointwise shared information.

### Appendix G.1. Probability Mass Exclusion

Suppose that *S* is a source and *T* is the target. The local mutual information for the realisations S=s and T=t is [8]:

(A22) $$i\left(t;s\right)=\log\frac{\mathrm{Pr}(T=t|S=s)}{\mathrm{Pr}(T=t)}\equiv \log\frac{\mathrm{Pr}(T=t,S=s)}{\mathrm{Pr}(S=s)}-\log\mathrm{Pr}\left(T=t\right).$$

We may write:

$$\begin{array}{cc} \mathrm{Pr}(T=t) & =p(s,t)+\mathrm{Pr}(T=t\;\;\mathrm{and}\;\;S\neq s),\\ \mathrm{Pr}(S=s) & =1-\mathrm{Pr}(S\neq s). \end{array}$$

Therefore, the local mutual information may be written as:

(A23) $$i\left(t;s\right)=\log\frac{\mathrm{Pr}(T=t)-\mathrm{Pr}(T=t\;\;\mathrm{and}\;\;S\neq s)}{1-\mathrm{Pr}(S\neq s)}-\log p\left(T=t\right).$$

Hence, forming the conditional probability Pr(T=t|S=s) in (A22) can be conceptualised as happening in two steps. The first is to exclude the probability mass Pr(T=tandS≠s) in the numerator and then to rescale the probability by dividing by 1−Pr(S≠s). The notion of *probability mass exclusion* is due to Finn and Lizier [67] and it is used in the formulation of the Isx method. This basic argument can be extended to the case of two or more input sources [8]. For two sources, we have:

(A24) $$i\left(t;s_{1},s_{2}\right)=\log\frac{\mathrm{Pr}\left(T=t\right)-\mathrm{Pr}(T=t\;\;\mathrm{and}\;\;\left\{S_{1}\neq s_{1}\;\;\mathrm{or}\;\;S_{2}\neq s_{2}\right\})}{1-\mathrm{Pr}(S_{1}\neq s_{1}\;\;\mathrm{or}\;\;\;S_{2}\neq s_{2})}-\log P\left(T=t\right).$$

### Appendix G.2. Shared Exclusions

From [8], the idea now is that shared information should be linked to *shared* exclusions of probability mass, i.e., possibilities being excluded redundantly by all joint sources, e.g., the exclusions induced by both $S_{1}\neq s_{1}$ and $S_{2}\neq s_{2}$. This suggests the removal of the intersection of the events $S_{1}\neq s_{1}$ and $S_{2}\neq s_{2}$ (rather than their union), and subsequent rescaling [8]. This leads to the definition of pointwise shared information (Shd${}^{p}$) as:

(A25) $$\begin{array}{cc} {\mathrm{Shd}}^{p} & :=\log\frac{\mathrm{Pr}\left(T=t\right)-\mathrm{Pr}(T=t\;\;\mathrm{and}\;\left\{S_{1}\neq s_{1}\;\;\mathrm{and}\;\;S_{2}\neq s_{2}\right\})}{1-\mathrm{Pr}(S_{1}\neq s_{1}\;\;\mathrm{and}\;\;S_{2}\neq s_{2})}-\log\mathrm{Pr}\left(T=t\right)\\ \; & \equiv \log\frac{\mathrm{Pr}(T=t\;\;\mathrm{and}\;\left\{S_{1}=s_{1}\;\;\mathrm{or}\;\;S_{2}=s_{2}\right\})}{\mathrm{Pr}(S_{1}=s_{1}\;\;\mathrm{or}\;\;S_{2}=s_{2})}-\log\mathrm{Pr}\left(T=t\right), \end{array}$$

which may be split into informative (Shd${}^{+}$) and misinformative (Shd${}^{-}$) components:

(A26) $$\begin{array}{cc} {\mathrm{Shd}}^{+} & :=\log\frac{1}{1-\mathrm{Pr}(S_{1}\neq s_{1}\;\;\mathrm{and}\;\;S_{2}\neq s_{2})}\equiv \log\frac{1}{\mathrm{Pr}(S_{1}=s_{1}\;\;\mathrm{or}\;\;S_{2}=s_{2})}, \end{array}$$

(A27) $$\begin{array}{cc} {\mathrm{Shd}}^{-} & :=\log\frac{\mathrm{Pr}(T=t)}{\mathrm{Pr}\left(T=t\right)-\mathrm{Pr}\left(T=t\;\;\mathrm{and}\;\;S_{1}\neq s_{1}\;\;\mathrm{and}\;\;S_{2}\neq s_{2}\right)},\\ \; & \equiv \log\frac{\mathrm{Pr}(T=t)}{\mathrm{Pr}(T=t\;\;\mathrm{and}\;\;\left\{S_{1}=s_{1}\;\;\mathrm{or}\;\;S_{2}=s_{2}\right))}, \end{array}$$

and:

$${\mathrm{Shd}}^{p}={\mathrm{Shd}}^{+}-{\mathrm{Shd}}^{-}.$$

We illustrate only the average Isx PID here, but full pointwise PIDs are available, both for the informative and the misinformative components; see [8].

### Appendix G.3. Application of the Isx Method to the AND Distribution

We now apply this to the AND probability distribution, defined in Table A1. We consider each realisation in turn and compute the pointwise measure of information along with its informative and misinformative components.

#### Realisation (0,0,0)

Referring to Table A1, the exclusions induced by the event $S_{1}=0$ are (1,0,0) and (1,1,1), and those induced by the event $S_{2}=0$ are (0,1,0) and (1,1,1). Hence, the shared exclusion induced by both events $S_{1}=0$ and $S_{2}=0$ is (1,1,1). So, excluding (1,1,1), and rescaling by 1–1/4 gives a probability of 1/3 to each of the three remaining realisations. The conditional probability that T=0, given that $S_{1}=0$ or $S_{2}=0$ is then equal to 1, since all three realisations include the event T=0. This exclusion (1,1,1), is therefore an informative exclusion, since its exclusion from the sample space increases the probability that T=0 from its prior value of 0.75 to the value 1.

Using (A25)–(A27), we consider the informative component, Shd${}^{+}$.

$$\mathrm{Pr}\left(S_{1}\neq 0\;\;\mathrm{and}\;\;S_{2}\neq 0\right)=\mathrm{Pr}\left(S_{1}=1\;\;\mathrm{and}\;\;S_{2}=1\right)=\frac{1}{4}.$$

Therefore,

$${\mathrm{Shd}}^{+}=\log\frac{1}{1-\frac{1}{4}}=\log\frac{4}{3}.$$

For the misinformative component, Shd${}^{-}$, we note that $\mathrm{Pr}\left(T=0\right)=\frac{3}{4}$ and:

$$\mathrm{Pr}\left(T=0\;\;\mathrm{and}\;\;S_{1}\neq 0\;\;\mathrm{and}\;\;S_{2}\neq 0\right)=\mathrm{Pr}\left(T=0,S_{1}=1,S_{2}=1\right)=0,$$

since the realisation (1,1,0) has probability 0. Therefore Shd${}^{-}$ = 0, and so the pointwise shared information contributed by realisation (0,0,0) is:

$${\mathrm{Shd}}^{p}=\log\frac{4}{3}.$$

#### Realisation (0,1,0)

Referring to Table A1, the exclusions induced by the event $S_{1}=0$ are (1,0,0) and (1,1,1), and those induced by the event $S_{2}=1$ are (0,0,0) and (1,0,0). Hence, the shared exclusion induced by both events $S_{1}=0$ and $S_{2}=0$ is (1,0,0). So, excluding (1,0,0), and rescaling by 1–1/4 gives a probability of 1/3 to each of the remaining realisations. The conditional probability that T=0, given that $S_{1}=0$ or $S_{2}=1$, is then equal to 2/3, since two of the remaining realisations include the event T=0. The exclusion (1,0,0) is therefore a misinformative exclusion, since its exclusion from the sample space decreases the probability that T=0 from its prior value of 0.75 to the lower value of 2/3, while for this realisation, we know that the event T=0 has happened.

Using (A25)–(A27) we consider the informative component, Shd${}^{+}$.

$$\mathrm{Pr}\left(S_{1}\neq 0\;\;\mathrm{and}\;\;S_{2}\neq 1\right)=\mathrm{Pr}\left(S_{1}=1\;\;\mathrm{and}\;\;S_{2}=0\right)=\frac{1}{4}.$$

Therefore,

$${\mathrm{Shd}}^{+}=\log\frac{1}{1-\frac{1}{4}}=\log\frac{4}{3}.$$

For the misinformative component, Shd${}^{-}$, we note that $\mathrm{Pr}\left(T=0\right)=\frac{3}{4}$ and:

$$\mathrm{Pr}\left(T=0\;\;\mathrm{and}\;\;S_{1}\neq 0\;\;\mathrm{and}\;\;S_{2}\neq 1\right)=\mathrm{Pr}\left(T=0,S_{1}=1,S_{2}=0\right)=1/4,$$

therefore,

$${\mathrm{Shd}}^{-}=\log\frac{\frac{3}{4}}{\frac{3}{4}-\frac{1}{4}}=\log\frac{3}{2}$$

and so, the pointwise shared information contributed by realisation (0, 0, 0) is:

$${\mathrm{Shd}}^{p}=\log\frac{4}{3}-\log\frac{3}{2}=-\log\frac{9}{8}.$$

This means that the net pointwise contribution is misinformative.

The remaining values are given in Table A5.

**Table A5:** Pointwise calculations for application of the Isx method to the AND distribution.

$\left(s_{1},s_{2},t\right)$	Shd${}^{+}$	Shd${}^{-}$	Shd${}^{p}$
(0,0,0)	$\log\frac{4}{3}$	0	$\log\frac{4}{3}$
(0,1,0)	$\log\frac{4}{3}$	$\log\frac{3}{2}$	$-\log\frac{9}{8}$
(1,0,0)	$\log\frac{4}{3}$	$\log\frac{3}{2}$	$-\log\frac{9}{8}$
(1,1,1)	$\log\frac{4}{3}$	0	$\log\frac{4}{3}$

Computing the average of the pointwise shared components with respect to the AND distribution, we obtain:

$$\mathrm{Shd}=\frac{1}{4}\log\frac{4}{3}-\frac{1}{4}\log\frac{9}{8}-\frac{1}{4}\log\frac{9}{8}+\frac{1}{4}\log\frac{4}{3}=\frac{1}{2}\log\frac{32}{27}≐0.1226.$$

Therefore, using (A1)–(A3) and (A5)–(A7), we have:

$$\begin{array}{ccc} \mathrm{Unq}S_{1} & =I(T;S_{1})-\mathrm{Shd}≐0.3113-0.1126 & ≐0.1887,\\ \mathrm{Unq}S_{2} & =I(T;S_{2})-\mathrm{Shd}≐0.3113-0.1126 & ≐0.1887,\\ \mathrm{Syn} & =I\left(T;S_{1},S_{2}\right)-I\left(T;S_{2}\right)-\mathrm{Unq}S_{1}≐0.8113-0.3113-0.1887 & ≐0.3113. \end{array}$$

Thus, we have found the Isx PID.

## Appendix H. Comparison of the Results

The results are combined in Table A6.

**Table A6:** The PID components obtained by applying six methods to the AND probability distribution.

	PID					
	Imin	Ibroja	Idep	Iccs	Ipm	Isx
Unq$S_{1}$	0	0	0.2296	0.2075	−0.2497	0.1887
Unq$S_{2}$	0	0	0.2296	0.2075	−0.2497	0.1887
Shd	0.3113	0.3113	0.0817	0.1038	0.5613	0.1226
Syn	0.5000	0.5000	0.2704	0.2925	0.7497	0.3113

For the AND distribution, we see that the Isx method produces components that are fairly similar to those given by the Idep and Iccs methods. These three methods produce very different results to those obtained using the other methods. In particular, the Isx results are very different to those of the Ipm method, and the Imin and Ibroja PIDs suggest that no unique information is transmitted.

## Appendix I. Further Comparison of PIDs

Using the data considered in Section 3.1 [28], we provide comparisons of four additional PIDs, Imin, Iproj, Iig, Iprec, along with Iccs and Idep. The probability distributions are of size 4 by 4 by 3, as before. When applying the the Iprec PID, it was found that no results were forthcoming after several hours, but Artemy Kolchinsky responded by kindly providing an amended version of his code [50], although it provides only a lower bound for the measure of redundancy. The components of the seven PIDs are plotted in Figure A3.

We consider first, the results obtained in the Control condition. The Imin, Iproj and Ibroja provide similar results for UnqB, while the values for the other four PIDs are similar to each other and generally larger than those for Imin, Iproj and Ibroja, with less variability. A curious feature of the of the UnqA plots is that Imin is zero for almost all of the data sets, perhaps indicating that it behaves like a minimum mutual information PID with these data. The values of UnqA given by Iccs, Idep and Iprec are more variable than the other PIDs, and larger on average. The shared information is generally higher for the Imin, Iproj and Ibroja PIDs, while Iccs, Idep and Iig show less variation. The synergy values are generally similar for all seven PIDs, but slightly higher on average for Imin, Iproj and Ibroja. When Baclofen is present, the values for UnqB, Shd and Syn are generally very similar for all seven PIDs; UnqA again is zero for Imin, and Iccs has some negative values. The statistics provided in Table A7 give numerical expression to these observations of the plots in Figure A3.

As in Section 3.1.3, we consider the within-neuron differences in the PID components. Summary statistics are available in Table A8, and the corresponding plots are in Figure A4. In Figure A4 we see that, apart from one neuron, all seven PIDs have a positive value for UnqB and a negative value for Syn, indicating an increase in UnqB and a decrease in Syn when Baclofen is present. Apart from a few neurons, UnqA is negative and Shd negative for all seven PIDs. The numerical summaries in Table A8 show that the seven PIDs fall, on average, into two groups. Imin, Iproj and Ibroja tend to suggest, on average, a larger increase in unique information due to the basal input and a larger decrease in shared information and synergy than do the other four PIDs: Iccs, Idep, Iig and Iprec.

In Section 3.1.4, the results of significance tests were presented. If a further four researchers were each to use one of Imin, Iproj, Iig or Iprec, and then apply a statistical test of the null hypothesis that the median value of synergy is the same in the absence and in the presence of Baclofen, then each of them would find a statistically significant reduction, on average, in the synergy component (all *p*-values are less than 0.001). If the four researchers were also to apply a test concerning the shared information component, they would again reach the same statistical conclusion, using Imin (P < 0.001), Iproj (P < 0.001), Iig (P < 0.001) and Iprec (P = 0.03), that there is a reduction, on average, in the shared information in the presence of Baclofen. They, like the researchers who used Ibroja, Iccs and Idep in Section 3.1.3, would reach the same statistical conclusion.

**Table A7:** Physiological L5b neuronal recording data. Summary statistics for 15 neurons. PID components are shown for each PID method and each experimental condition. The sample median (Md) and the sample quartiles (q${}_{L}$, q${}_{U}$) are stated as percentages of the joint mutual information.

		Control							Baclofen						
		**Imin**	**Iproj**	**Ibroja**	**Iccs**	**Idep**	**Iig**	**Iprec**	**Imin**	**Iproj**	**Ibroja**	**Iccs**	**Idep**	**Iig**	**Iprec**
	q${}_{L}$	33.3	33.5	33.7	41.9	43.7	44.0	43.4	64.7	65.2	65.7	66.4	67.9	68.5	66.4
UnqB	Md	44.6	49.2	49.7	55.0	56.1	50.3	53.2	74.5	74.5	74.5	73.9	76.3	75.5	74.7
	q${}_{U}$	60.7	60.7	60.8	61.1	65.7	68.6	61.7	80.1	81.3	82.0	79.5	84.3	82.8	82.8
	q${}_{L}$	0.0	0.1	0.2	1.7	6.1	2.0	1.4	0.0	0.0	0.1	-0.7	1.9	0.4	0.5
UnqA	Md	0.0	0.4	0.7	6.7	10.3	4.8	2.7	0.0	0.3	0.7	-0.1	4.0	0.6	1.7
	q${}_{U}$	0.0	2.4	5.3	11.4	13.6	8.2	11.7	0.0	1.1	1.9	1.2	5.0	1.2	2.7
	q${}_{L}$	11.8	11.0	9.4	6.4	3.4	6.4	3.7	4.3	3.7	3.5	3.7	1.5	3.7	2.8
Shd	Md	16.3	13.7	11.9	8.7	5.4	9.5	6.6	5.2	5.1	5.0	4.7	2.7	4.0	3.8
	q${}_{U}$	19.3	17.4	17.1	10.1	6.6	10.9	11.4	8.0	6.8	5.5	7.6	3.2	5.2	4.8
	q${}_{L}$	30.2	27.5	25.9	25.1	19.0	22.9	24.4	13.8	12.9	12.7	16.4	11.3	12.9	12.2
Syn	Md	38.8	38.8	38.6	33.9	32.0	32.6	30.0	22.2	21.7	21.1	21.6	17.0	17.2	19.8
	q${}_{U}$	47.7	46.4	44.4	37.3	36.0	38.4	37.8	28.4	27.6	27.2	27.7	24.7	22.7	26.1

**Table A8:** Physiological L5b neuronal recording data. Summary statistics of within-neuron differences for 15 neurons. The sample median (Md) and the sample quartiles (q${}_{L}$, q${}_{U}$) of the differences (taken as Baclofen minus Control) are provided for each PID component.

		Imin	Iproj	Ibroja	Iccs	Idep	Iig	Iprec
	q${}_{L}$	21.8	21.7	21.7	14.7	16.9	16.8	16.0
UnqB	Md	27.9	28.6	26.6	22.1	22.1	20.4	21.4
	q${}_{U}$	33.3	30.3	28.3	23.4	24.2	26.8	26.9
	q${}_{L}$	0.0	−1.4	−2.6	−8.9	−9.0	−6.6	−9.7
UnqA	Md	0.0	−0.1	−0.3	−6.8	−6.0	−3.5	−1.4
	q${}_{U}$	0.0	0.0	0.0	−5.1	−4.0	−1.5	−0.5
	q${}_{L}$	−11.9	−10.8	−10.3	−3.6	−3.4	−5.9	−7.7
Shd	Md	−9.3	−9.2	−7.4	−2.4	−2.7	−4.1	−3.3
	q${}_{U}$	−7.4	−6.4	−4.6	−1.8	−1.8	−3.4	0.2
	q${}_{L}$	−20.7	−20.2	−19.0	−14.4	−13.0	−16.0	−14.9
Syn	Md	−19.1	−16.7	−14.9	−9.9	−11.1	−10.6	−12.0
	q${}_{U}$	−13.4	−13.4	−12.6	−6.8	−7.5	−8.3	−8.1
